# A compilation and characterisation of lithics in kimberlite and common maar-diatremes and tephra ring deposits

**DOI:** 10.1038/s41598-021-03307-7

**Published:** 2021-12-14

**Authors:** M. K. Fitzgerald, J. D. L. White

**Affiliations:** Department of Geology, University of Otago, Dunedin, 9016 New Zealand

**Keywords:** Geology, Volcanology

## Abstract

Maar-diatreme volcanoes are the second-most common type on land, occurring in volcanic fields within all major tectonic environments. Their deposits typically contain an abundance of lithic fragments quarried from the substrate, and many contain large, deep-sourced lithic fragments that were erupted to the surface. Primary volcaniclastic deposits fill the diatreme structure formed during eruption. There is negligible inelastic deformation of diatreme-adjacent country rock, indicating that country rock is removed to create the diatreme structures, either by being shifting downward below observable levels, ejected upward to contribute to surficial deposits, or dissolved and hidden in magma erupted or intruded at depth. No previous study has systematically reviewed and analysed the reported lithic fragments of maar-diatreme systems. We present a comprehensive compilation from published work of lithic characteristics in maar ejecta rings and in diatreme deposits of both common and kimberlite maar-diatremes. For maar-diatremes and their tephra ring deposits, we find no correlations among lithic clast sizes, shapes, depositional sites, and excavation depths. This is difficult to reconcile with models involving systematic diatreme deepening coupled with tephra-ring growth, but consistent with those involving chaotic explosions and mixing. Larger amounts of data are needed to further examine how these types of volcanoes operate.

## Introduction

Maar-diatreme volcanoes are among the most common volcanoes in the world^1^. Commonly situated within volcanic fields near many communities, their eruptions represent hazards to human populations across the globe^1^. We view kimberlite fields as a subset of volcanic fields and include kimberlite diatremes and maars throughout this paper except where we specifically distinguish them from "common" maars and maar-diatremes.

Unlike scoria cones, shield volcanoes, or composite volcanoes, common maar-diatreme volcanoes are broadly thought to be formed when magma interacts with groundwater at depth and causes subsurface explosions, which in turn fracture the surrounding host rock and pave the way for magma, juvenile products, and liberated country rock (lithics) to be propelled upwards and out of the newly formed subterranean volcanic structure^2–4^. From the Greek δία ("dia”—"through") and τρῆμα ("trema”—"hole, aperture"), the word diatreme is used to describe the structure, filled with juvenile fragments and lithic fragments, left by this process^5^. Explosions and eruptions create this structure within the pre-eruptive strata while also depositing juvenile material and host rock lithic fragments in the ejecta ring on the surface^3,5^. The term “ejecta ring” is used hereinafter as an encompassing term for ejecta rims, tuff rings, and tephra aprons.

**Figure 1:**
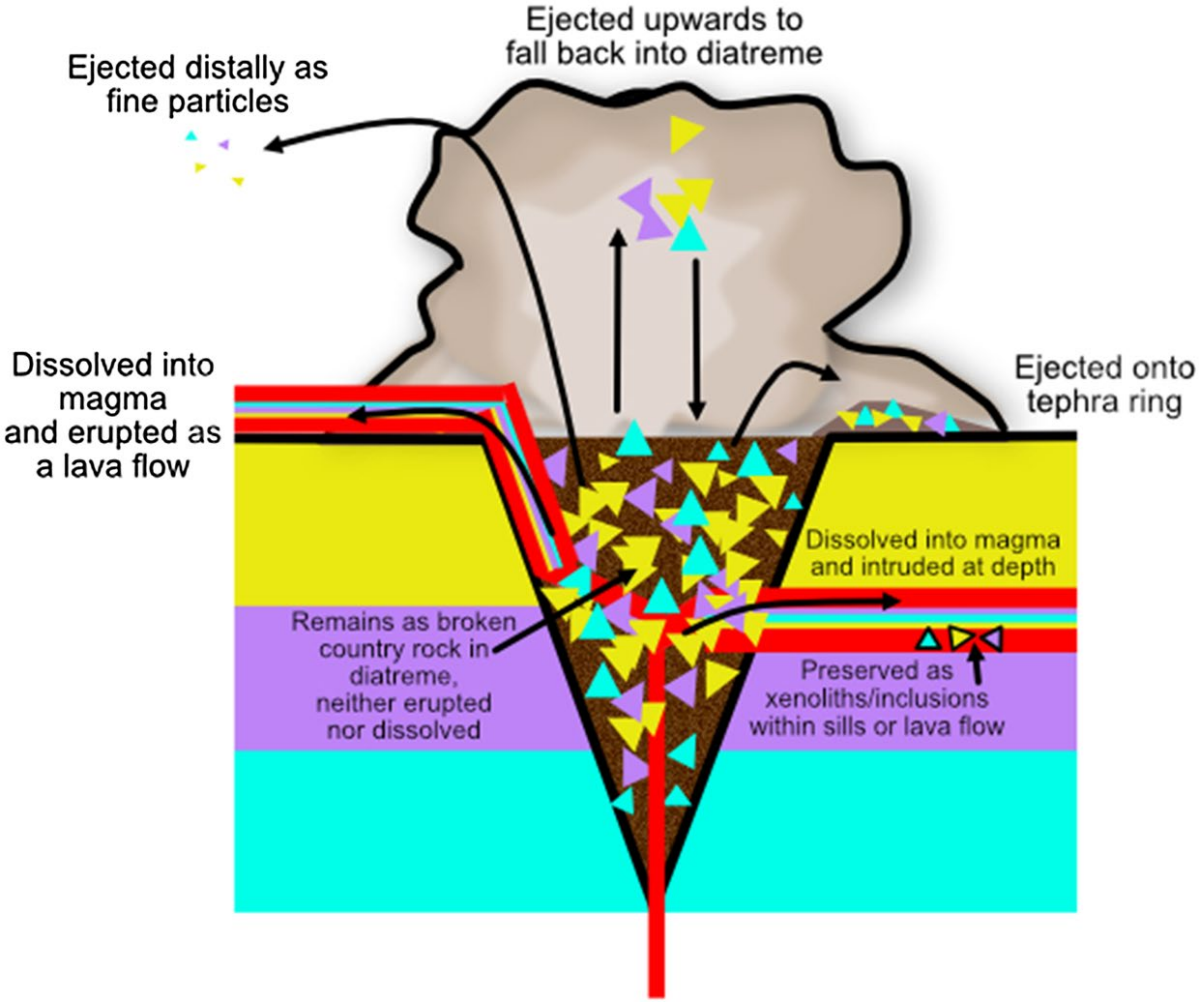
Illustration of possible trajectories for perturbed country rock in a maar-diatreme system. Disrupted country rock must be adequately accounted for as material either in the tephra ring, the newly formed diatreme, or as xenolith content in, or dissolved constituent of, magma that has been either erupted or focused into dikes and sills. Country rock that is ejected upwards and falls back into the diatreme does not leave a cavity; instead, it refills the diatreme with assorted broken/milled country rock fragments.

Diatreme deposits are found in space once occupied by country rock. There is no evidence that space has been made by inelastic deformation of country rock into which diatremes are emplaced^6,7^, so country rock must have been removed to produce the diatreme structures. Geometrically the country rock now missing, in the volume occupied by the diatreme deposits, could either be dropped downward, below levels of observation, ejected upward, to form parts of the ejecta ring or other surficial deposits, or hidden by dissolution into magma that is erupted or intruded at depth (Fig. 1).

Many kimberlite pipes, which host diamonds, are also diatreme structures^8^, but there is disagreement about whether or not they form by the same processes as do the more-common diatremes of mafic and even felsic magmas^9^. Field studies of kimberlite diatremes have yielded several models, including a “shared” one considered applicable for both kimberlite and common diatremes^10^, and another, in which uniquely volatile-rich magma^8^ forms a sustained eruptive jet often associated with wholesale fluidization within the diatreme^11^. In the “shared” model for kimberlite diatremes, explosions are traditionally considered (e.g.,^12,13^) to occur only at the base of the diatremes, which grow through a deepening progression of explosions governed by retreat of a cone of depression in the host aquifer.

Over the past couple of decades, modifications of the progressive-deepening model, applicable to both common and kimberlite diatremes, have been suggested^4,5,14–18^. Kimberlite-specific models have also been revisited^10,19–25^. Despite this abundance of work, however, we still lack a clear understanding of how large, deep-sourced host rock lithics are transported to the surface, and what patterns of lithic distribution reveal of diatreme-forming processes. In part, this is because even though the abundance of maar-diatremes, including mined or drilled kimberlites, provides ample opportunity to study their deposits, volcanic fields exposing diatreme deposits commonly lack corresponding surface structures^26^. With few exceptions^3,21^, studies of these volcanoes generally tend to focus on only one aspect of the overall structure—either the tephra ring or the diatreme infill.

The known ranges of lithic source depths and rock types offer great opportunities for using patterns of lithic fragment distribution and modification (e.g., abrasion) to help elucidate diatreme- and tephra ring-forming mechanisms. Data compiled here from published studies strongly suggest that there are no systematic patterns in the locations or characteristics of the lithics found within diatremes and tephra rings. This may indicate that ~ random stochastic processes play controlling roles in maar-diatreme eruptions, but larger amounts of data, more consistently acquired and reported, are needed to test this hypothesis. Other models suggest chaotic distributions^16,27,28^ of lithic transport and deposition, which are consistent with the existing dataset.

## Methods

To locate information for this compilation, major bibliographic databases were searched using the following terms: “lithics”, “diatremes”, “tephra”, “kimberlite”, “country rock”, “phreatomagmatism”, “maar-diatremes”, “maar ejecta”, “tephra apron”, “ejecta ring”, and “tephra rings”, as well as various combinations of these terms. Papers included have primary information reporting lithic fragments present in common maar-diatreme and kimberlite maar-diatreme tephra rings and/or diatreme infill. Peer-reviewed journal publications with complete text were the only articles included in this analysis.

**Figure 2:**
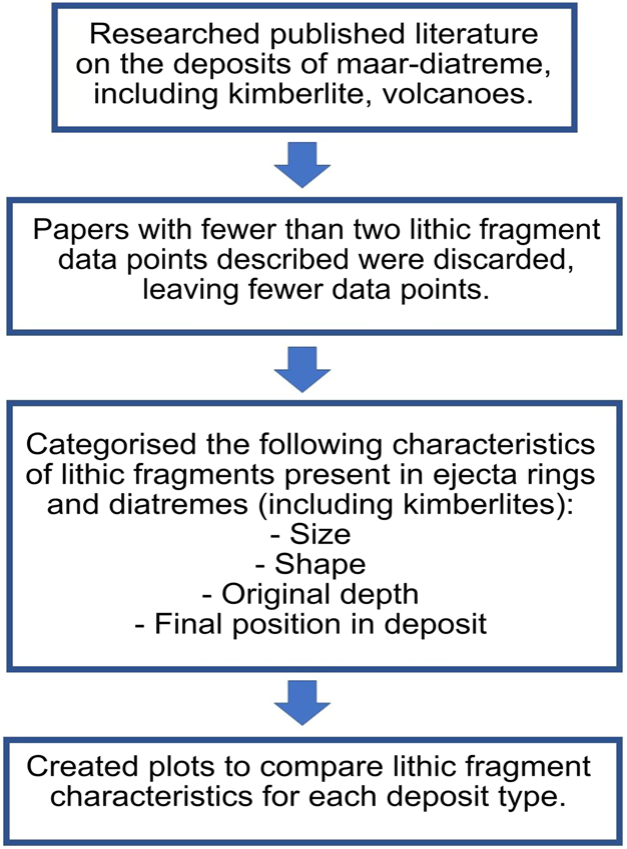
Brief flow diagram of methods used to extract relevant data from published literature for use in this study.

Descriptions of features were sought, with raw data recorded. Commonly reported characteristics were lithic sizes, shapes, original depths, and position in deposits, and these attributes were chosen in order to search for systematic patterns (Fig. 2). A total of 92 papers were examined, providing 160 deposit descriptions (see Supplementary Tables S1 and S2).

Through the 92 articles reviewed, there were various descriptions for the different aspects of the lithic fragment characteristics; therefore, categories for each of these features were created as follows.

*Sizes* Sizes were described with either specific measurements (metric or imperial units) or geological (generally Wentworth^29^) grain sizes, which were then converted to numeric values based on the grain size classes of White and Houghton^30^ (“fine lapilli” = 2–4 mm; “medium lapilli” = 4–16 mm; “coarse lapilli” = 16–64 mm; “block/bomb” =  > 64 mm). When reported simply as “lapilli”, grain sizes were assigned the value of 40 mm (the midpoint of White and Houghton’s lapilli classification), and “blocks” were assigned the value of 640 mm (the midpoint of White and Houghton’s medium block classification (Table 1)). If a range was given, the maximum value was used (within reason); where studies stated that most of the particles were, e.g., lapilli, with sparse decimetre-sized blocks throughout, the maximum lapilli size value was used to ensure recording of the size that was the most represented, rather than merely the absolute maximum block size present. All lithic fragment sizes were then converted to the Krumbein phi scale (φ), using Eq. (1):

1 $$\varphi \, = \, {-}\log 2\left( {{\text{D}}/{\text{D}}_{0} } \right)$$

**Table 1:** Block size distributions and corresponding phi values for categorization of lithic fragment sizes reported in literature.

	Min (mm)	Max (mm)	Phi min	Phi max
Small block	64	256	− 6	− 8
Medium block	256	1024	− 8	− 10
Large block	1024	4096	− 10	− 12
Megablock	4096	>	− 12	>

These terms are used in accordance with the inclination of volcanological researchers to use “block” instead of "boulder" for large clasts^51^.

where D represents the diameter of the grain size in mm, and D_0_ is a constant reference diameter of 1 mm^31^. Block ranges (“large”, “mega”, and “decimetre”) were assigned specific mm ranges according to sensible phi ranges (Table 1), when no specific numbers were available. If no size information was given at all, size was recorded as phi = 0 to allow plotting of the other characteristics of the respective deposit (plots with assigned “0” values for size are shown in Supplementary Figs. 1 and 2).

*Shape/angularity* Shape/angularity was either reported or inferred from published images, and assigned to one of six categories (angular, sub-angular, sub-angular to sub-rounded, sub-rounded, rounded, or unknown). If shape given ranged across more than one category, the midpoint of those categories was used, i.e., if the shape was “angular to sub-rounded”, it was recorded as “sub-angular”.

*Final position of lithics deposited in sequence* The final locations and characteristics of lithic fragments hold information that may assist in understanding the sequence of emplacement of diatremes and maar ejecta rings. For characterization of final position in sequence, the positions of lithics deposited in tephra rings were grouped as “upper”, “middle”, or “lower” tephra ring. For lithics described as “early” within literature, these were assigned to the lower tephra ring, and lithics described as “late” were assigned to the upper tephra ring. For diatremes, if position was not given, then “bedded” was considered to represent upper diatreme material, and “unbedded” was considered to represent lower diatreme material^5^.

*Original depth of lithic fragments* Original depths of the lithic fragments were recorded as either specific depths of original lithic position, or “shallow/deep” if the depths were not well-specified in the cited studies. Where lithics were from a known pre-eruptive sedimentary sequence, then it was possible to infer the original entrainment depth for the lithics. If depth was not given for a set of lithic fragments, yet their lithology was identical to a geological unit that was shown in a stratigraphical column, the midpoint of the depth for that unit was recorded as the original depth of the lithic fragments. Usually, ranges were given, therefore original depths of lithics were recorded as minimum and maximum. If specific depth was not given, nor could be inferred from stratigraphy, the following original lithic depths were assigned to the descriptions, interpolated from the other points given in the dataset: “shallow” = 0–200 m; “deep” = 200–700 m; “basement” = 200–500 m. If no depth information was given at all, maximum depth was recorded as 0 m, to allow plotting of the other characteristics of the respective deposit (plots with assigned “0” values for original depth are shown in Supplementary Figs. 1 and 2). If no maximum limit was given, the maximum original depth was recorded as the same as the minimum original depth. If no minimum limit was given, 0 m was used as the minimum depth.

## Results

We required that at least two characteristics (e.g., any two of “lithic size”, “lithic shape”, “final position of lithic”, and “original lithic depth”) be provided for results to be included for further analysis. Out of 160 deposits (across 92 papers) fitting this requirement, 77 deposits contained all four pieces of information sought. A total of 152 (95%) gave size information, 104 (65%) gave shape information, 153 (95.6%) gave the final position in the deposit, and 127 (79.4%) gave original depth information (Table 2).

**Table 2:** Total numbers, for each of the deposits suitable for use in this study, of specific lithic fragment observations available for each deposit type.

Deposit type	Number of deposits with at least two lithic characteristics reported	Number of deposits reporting lithic size	Number of deposits reporting lithic shape	Number of deposits reporting final position of lithic fragment in sequence	Number of deposits reporting original depth of lithic fragment (E denotes exact depth or stratigraphy provided; I denotes inferred from information in study)	Number of deposits with all four lithic characteristics provided
Common tephra ring	68	67 (99%)	40 (59%)	68 (100%)	59 (35E, 24I) (87%)	31 (46%)
Kimberlite tephra ring	5	4 (80%)	4 (80%)	5 (100%)	4 (4E) (80%)	2 (40%)
***Tephra ring total***	***73***	***71 (97%)***	***44 (60%)***	***73 (100%)***	***63 (39E, 24I) (86%)***	***33 (45%)***
Common diatreme infill	47	44 (94%)	32 (68%)	42 (89%)	41 (17E, 24I) (87%)	25 (53%)
Kimberlite diatreme infill	40	37 (93%)	28 (70%)	38 (95%)	23 (12E, 11I) (58%)	19 (48%)
***Diatreme infill total***	***87***	***81 (93%)***	***60 (69%)***	***80 (92%)***	***64 (29E, 35I) (74%)***	***44 (51%)***
***Total***	***160***	***152 (95%)***	***104 (65%)***	***153 (96%)***	***127 (68E,59I) (79%)***	***77 (48%)***

Significant values in bolditalics.

**Figure 3:**
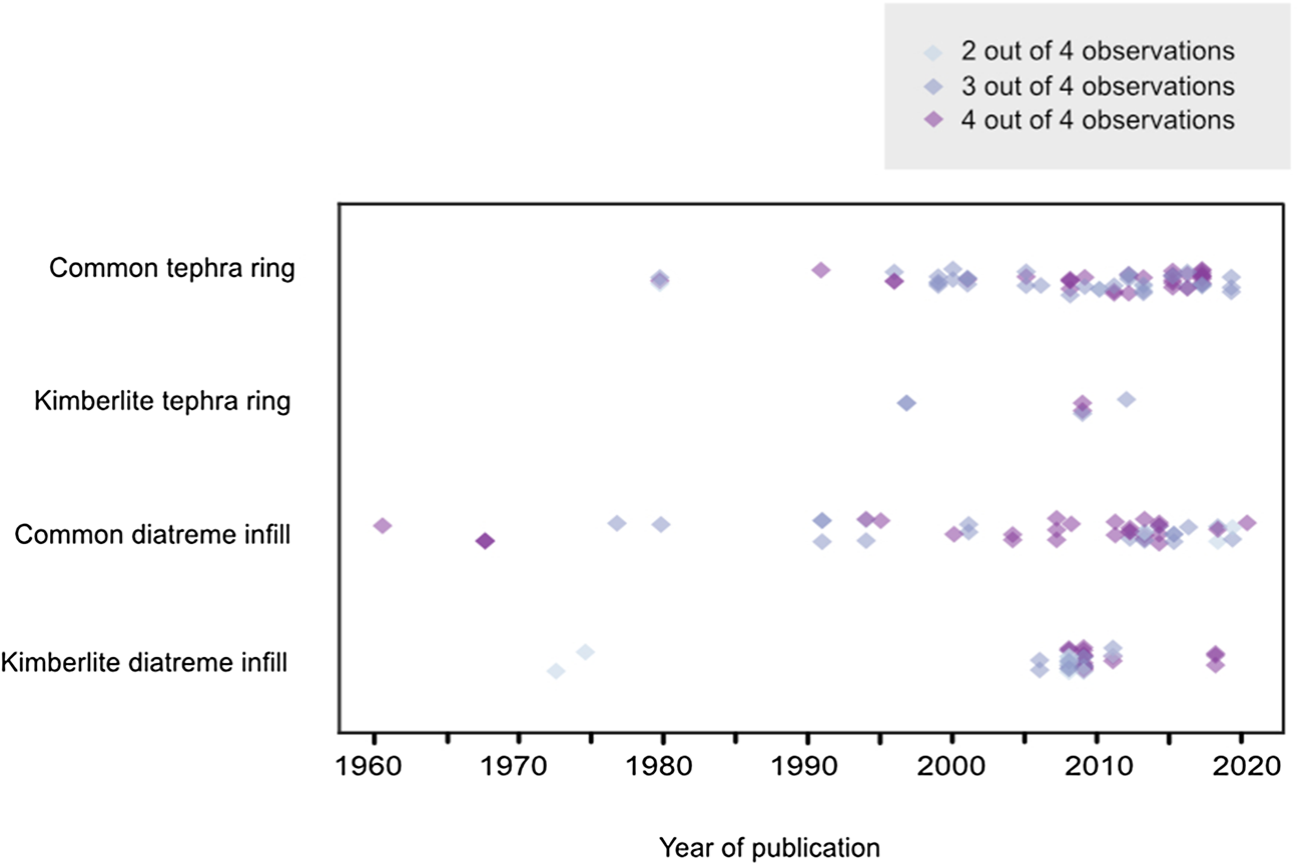
Number of applicable observations (lithic size, shape, original depth, or final position in sequence) provided per deposit, shown in relation to year published.

In terms of extracting data on the locations of lithic origin, sites of lithic deposition, abundance of lithics, size of lithics, and shapes of lithics, it was found that the more recently a paper was published, the larger the likelihood that it contained more information for further analysis (Fig. 3). It is likely that to test evolving diatreme models, researchers have increasingly recognized the role of lithic fragments as potential indicators of fragmentation sites and transport processes and histories.

Of the original depth observations, 53.5% were given in metres (or imperial units) or in specific stratigraphic context, and 46.5% were inferred from information gleaned regarding the published geological setting and lithic description. Table 2 shows the type of depth information available for each deposit type, given as E (exact) or I (inferred).

**Figure 4:**
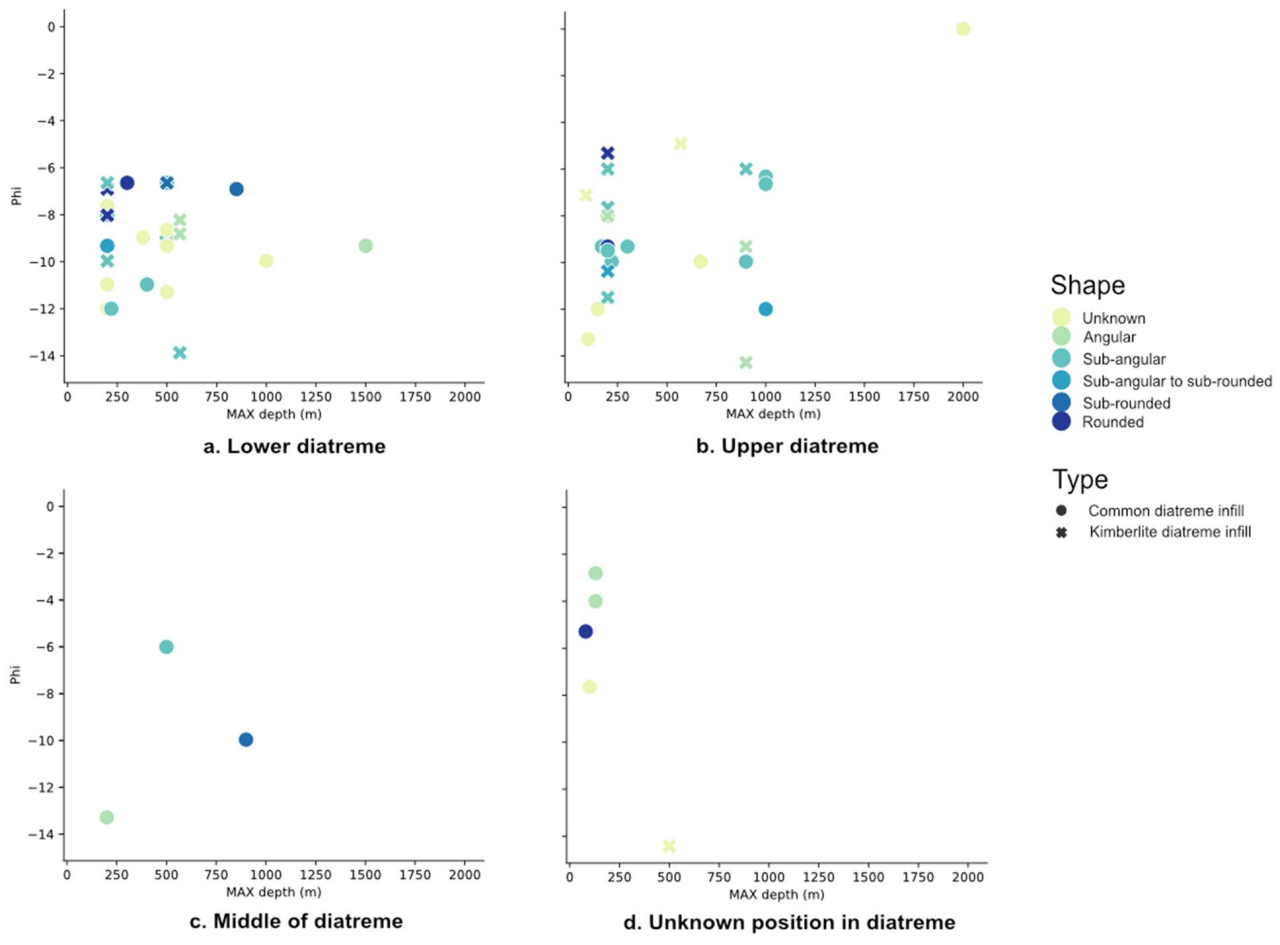
Each part of the diatreme infill sequence plotted to show final position in diatreme, maximum original depth, phi, and shape for lithics in both common maar-diatreme infill and kimberlite maar-diatreme infill deposits. (**a**) lower; (**b**) upper; (**c**) middle; (**d**) unknown diatreme area.

**Figure 5:**
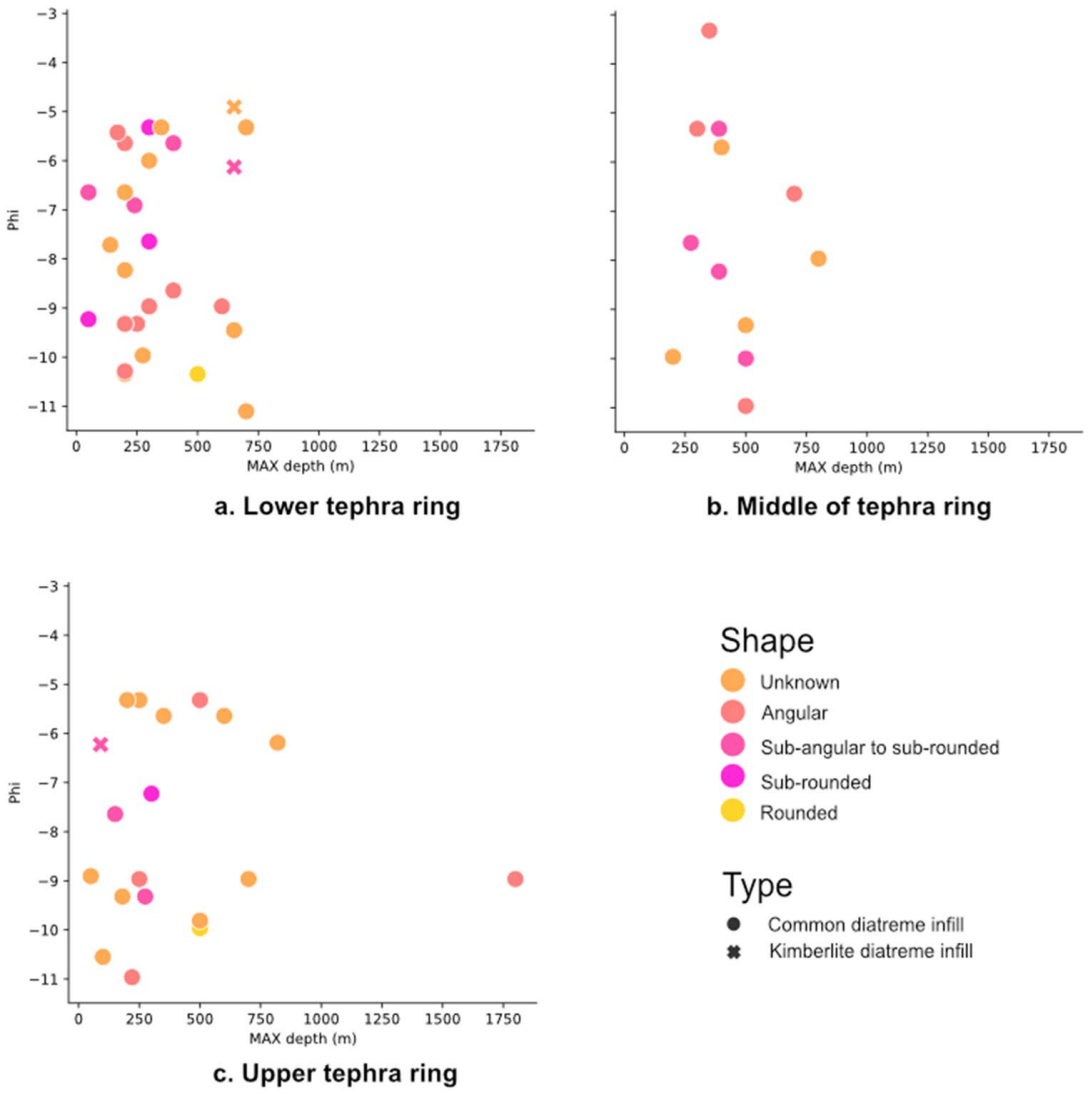
Each part of the tephra ring sequence plotted to show final position in tephra ring, maximum original depth, phi, and shape for lithics in both common maar-diatreme tephra ring and kimberlite maar-diatreme tephra ring deposits. (**a**) lower; (**b**) middle; (**c**) upper tephra ring.

Each part of the sequence in diatreme infill was plotted against maximum original depth, phi, and shape, for both common maar-diatreme and kimberlite maar-diatreme deposits. With all unknown “zero” values for size and original depth removed, no trend is visible for any of the attributes (Fig. 4). Each part of the sequence in tephra rings was plotted against maximum original depth, phi size, and shape, for both common maar-diatreme and kimberlite maar-diatreme tephra ring deposits. With all unknown “zero” values for size and original depth removed, no trend is visible for any of the attributes (Fig. 5).

## Discussion and conclusions

Country rock is necessarily removed to make a diatreme structure. The final locations and characteristics of lithic fragments hold information critically important for understanding the processes that take place during formation of diatremes and their associated surficial maar ejecta rings. The information can be used both to test existing models, and to enhance or replace them based on constraints provided by the lithic population.

**Figure 6:**
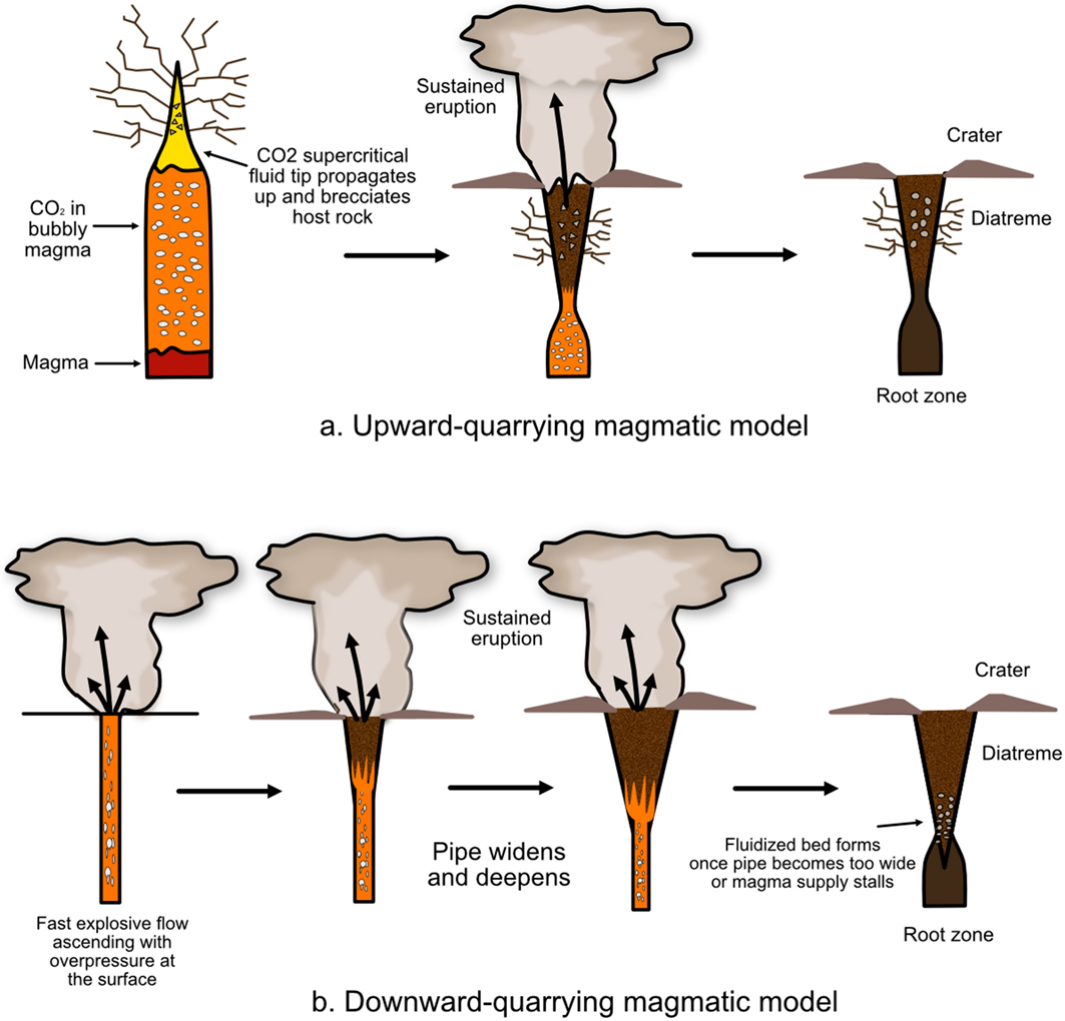
Magmatic models for maar-diatreme eruption. Current magmatic models for maar-diatreme volcanoes consider that magmatic fragmentation involves a volatile-rich magma travelling from depths through pre-eruptive stratigraphy to the surface. In the upward-quarrying model (**a**), ascent is accompanied by rapid exsolution and expansion of the volatiles within the magma, forming a CO_2_ fluid tip which causes brittle fragmentation of surrounding country rock under high strain rates during rapid ascent^20,37,38^. In the downward-quarrying model (**b**), magma quickly ascends to the surface under overpressure conditions, and ejection of material causes the pipe to deepen and widen with downward progression^11,23,39,40^.

**Figure 7:**
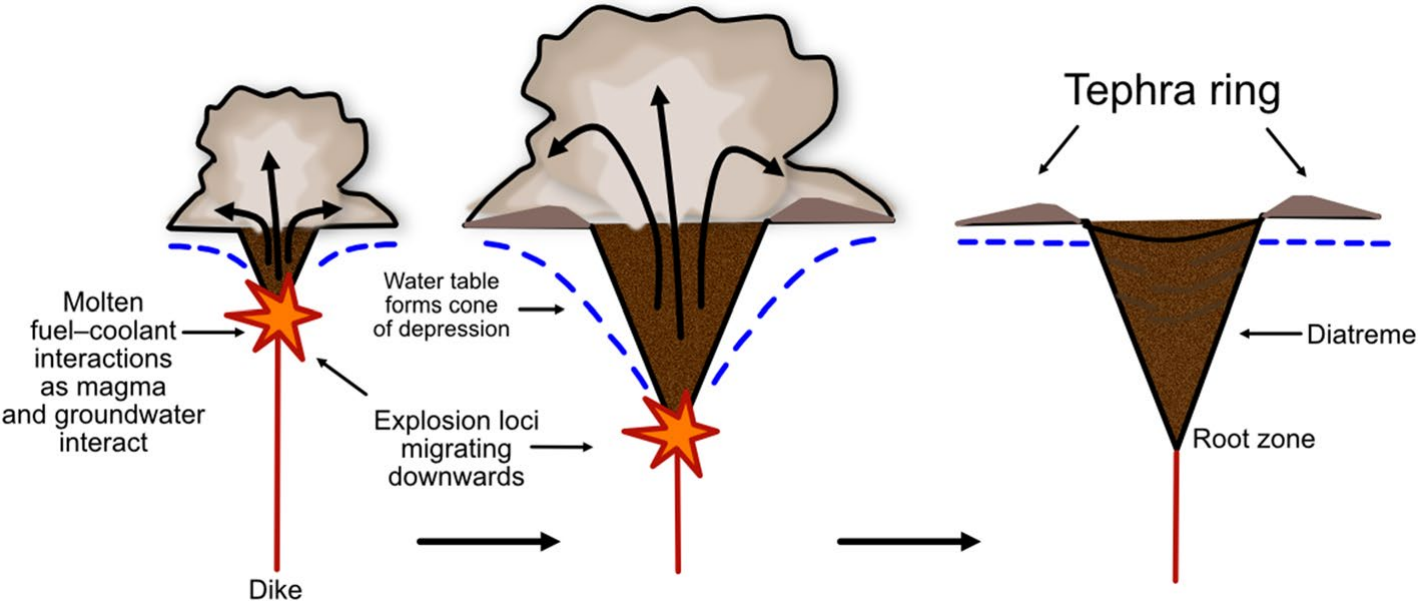
The original phreatomagmatic model for maar-diatreme eruption. In the original phreatomagmatic model for maar-diatreme eruptions, proposed by Lorenz^32^, explosions move downwards as limited amounts of groundwater are released explosively as steam, causing a downward-propagating excavation of country rock and formation of diatreme and tephra ring with subsequent explosions.

**Figure 8:**
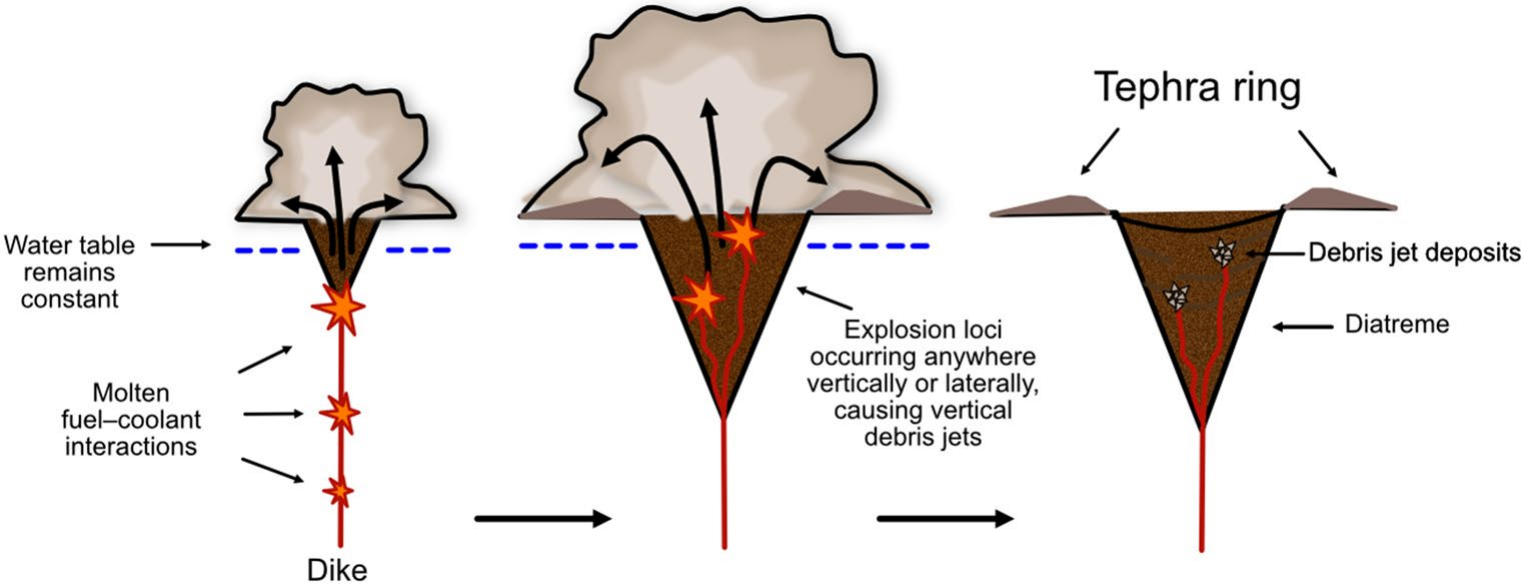
The revised phreatomagmatic model for maar-diatreme eruption. In the revised phreatomagmatic model for maar-diatremes, proposed by Valentine and White^14^, as magma rises up via a dike, the explosions can occur anywhere, provided the hydrostatic pressure does not exceed the critical pressure of water; explosions do not necessarily occur along a downward-progressing path. This creates a “proto-diatreme”, which hosts ongoing explosions with both vertically and laterally varying loci. Country rock and earlier-formed dikes are brecciated and then mixed within vertical jets of debris taking place within the diatreme, bolstered by subsidence of fallen pyroclasts and wall rock falling around the edges of the debris jets.

There are three existing model families that describe the mechanisms which form maar-diatremes. One family (Fig. 6) is considered unique to kimberlite and involves sustained magmatic carbon-dioxide jetting plus in-diatreme fluidization^8,11,19,22–25,32,33^ or a convective column^19,23,34^. Another (Fig. 7) is considered applicable to both common maar-diatremes and their kimberlite equivalents, with energy supplied by magmatic heat through phreatomagmatic explosions that track a retreating water table after eruption begins^10,12,13^. A third (Fig. 8) builds on features of the second, but involves discrete explosions at multiple sites and times, which may be, but are not necessarily, phreatomagmatic^4,5,14–18,35,36^. Our analysis here begins by using existing published lithic information to probe existing model families. We subsequently make a case for the sort of information needed from future studies to better employ lithic information in the overall volcanological analysis of maars and diatremes.

### Implications of compiled lithic populations for the magmatic kimberlite model

A "magmatic" eruption, for the purpose of this study, is referred to as one that takes place without involvement of water (ice, groundwater, or surface water in rivers, lakes, or oceans). Magmatic interaction with water results in the classification of an eruption as “phreatomagmatic” in this article. Explosive magmatic eruptions involve fragmentation of magma; they include episodic strombolian bursts and discrete Vulcanian explosions, as well as sustained fountaining to plinian ash columns.

**Figure 9:**
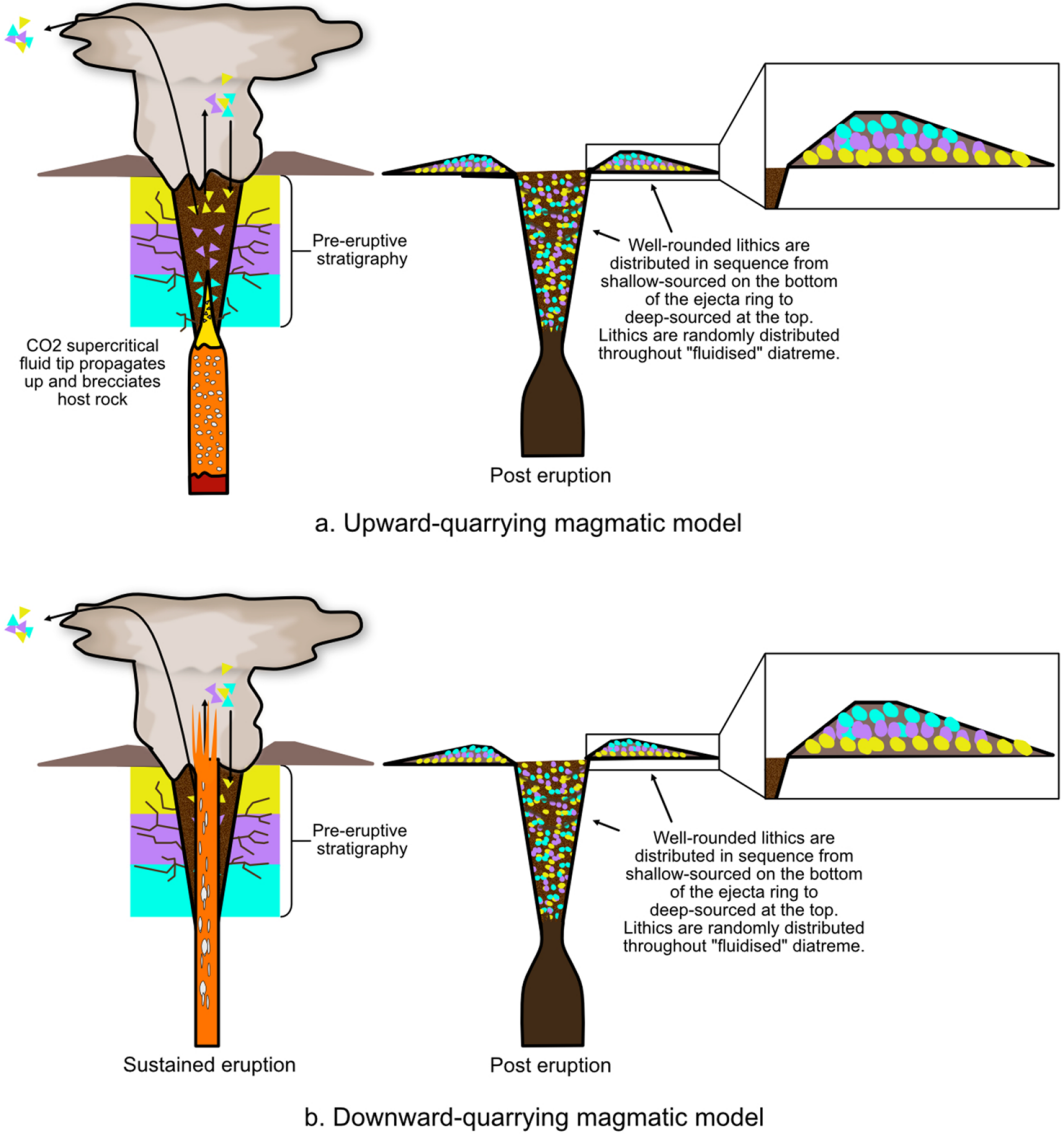
Lithic fragment behaviour expected in both upward- (**a**) and downward (**b**)-quarrying magmatic maar-diatreme eruptions. A chaotic distribution of lithic clasts in a diatreme is expected from a magmatic kimberlite eruption involving wholesale vent fluidization and mixing during a sustained eruption.

A downward-quarrying magmatic diatreme formation model involves the ejection of all fragments (juvenile and lithic) up to approximately 1 m in size as a rapidly moving gas-filled magma is subjected to near-vent overpressure^19^. This causes the pipe to widen and deepen, and once the pipe becomes too wide, or the magma supply falters, the gas velocity driving the eruption wanes, resulting in a fluidized bed of unerupted juvenile and lithic particles displaying a significant rounding of all particles and an absence of large lithics (Fig. 9b)^19^. The characteristics of the lithic fragments studied here do not support this, as there are a variety of sizes and shapes of lithics present within the lower diatreme deposits. However, this model is compatible with finding lithics from many different sources now present at the same depth, with a “homogenized” appearance.

In upward-quarrying magmatic models, degassing of ascending magma followed by a decompression front travelling back down the magma column results in the entrainment and ascent of wall-rock and mantle fragments with upwelling of magma^20,32,33^. Well-rounded, deep-seated xenoliths would occur in the tephra rings, increasing in size and abundance as the eruption sequence progressed^33^. There is no pattern within the tephra rings that supports an increase of deep-seated lithic fragments from the lower to the upper tephra ring (Fig. 9a), nor are lithic reports dominated by well-rounded fragments.

### Implications of compiled lithic populations for the common maar-diatreme model

In the original phreatomagmatic model for maar-diatreme eruptions, proposed by Lorenz, the magma’s thermal energy is transmuted into shock waves that fragment both the magma itself and the surrounding host country rock, while also converting groundwater from the surrounding host sedimentary sequence into steam, which carries the fragmented magma and country rock up to the surface^41^. The first fuel–coolant interaction (FCI)^42^ results in a diatreme, crater, and ring of ejecta (Fig. 7).

**Figure 10:**
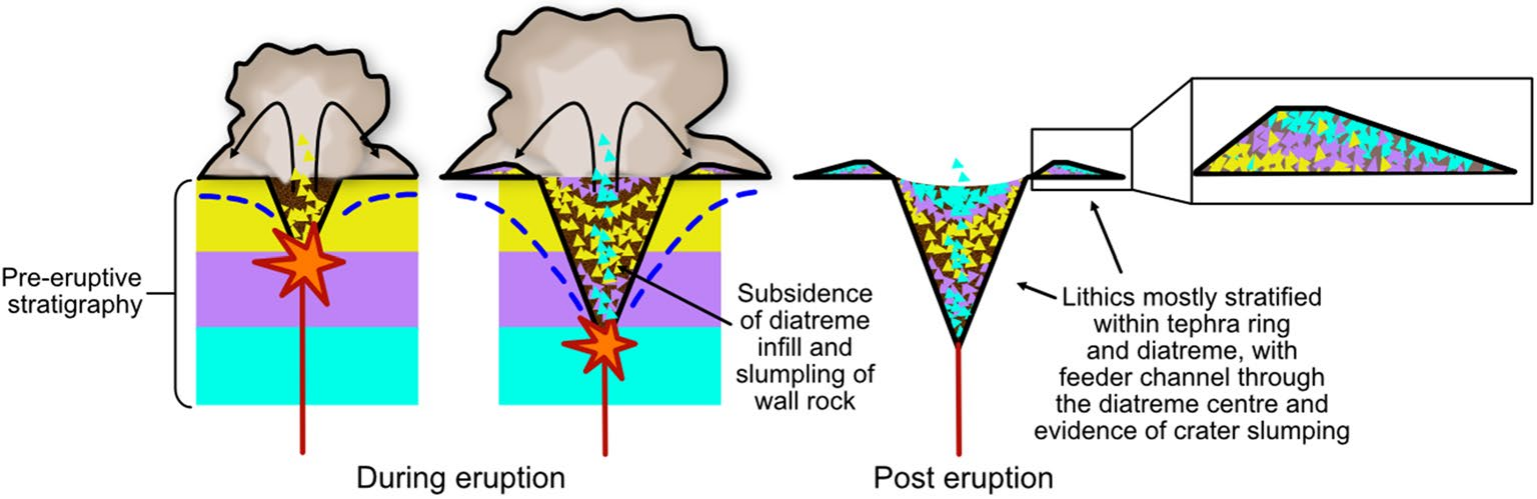
Lithic fragment behaviour expected in an original phreatomagmatic model maar-diatreme eruption. The progressive deepening of the explosion site lends itself to a systematic deepening of the deposited country rock lithic origins as the tephra ring sequence progresses through time. The result is that lithics liberated from shallow strata would arrive at the surface first and should dominate the lowest (earliest) units of the tephra ring (and the upper diatreme infill), while deep-seated lithics abound the upper (latest) units. Because the lithics are ejected in a single movement, the final resting place of a lithic is controlled by its depth of origin and the transport processes culminating in its emplacement into the tephra ring (or diatreme).

In this phreatomagmatic model, the progressive deepening of the explosion site lends itself to a systematic deepening of the deposited country rock lithic origins as the tephra ring sequence progresses through time. This suggests that lithics liberated from shallow strata should arrive at the surface first and dominate the lowest (earliest) units of the tephra ring (and the upper diatreme infill), while deep-seated lithics abound the upper (latest) units^4,41,43^ (Fig. 10). Because the lithics are ejected in a single movement, the final resting place of a lithic is controlled by its depth of origin and the transport processes culminating in its emplacement into the tephra ring (or diatreme)^44^.

This model fails to account for volume discrepancies between tephra ring material and crater and diatreme size, as well the as inconsistent emplacement of lithics in tephra rings found in this study. The dataset of reported lithic fragments preserved in tephra rings does not show any overall trend of progressive correlation with original depth, although studies for a few sites do report this pattern (e.g., Tihany Maar^45^ and Bea’s Crater^46^). Moreover, the conditions required to hold a steady magma influx while driving downward water table migration require increasing energy^13^, as does an eruptive pathway to be continually tunnelled through thick diatreme infill with deepening explosion loci^16,43^.

### Implications of compiled lithic populations for the revised common maar-diatreme model

**Figure 11:**
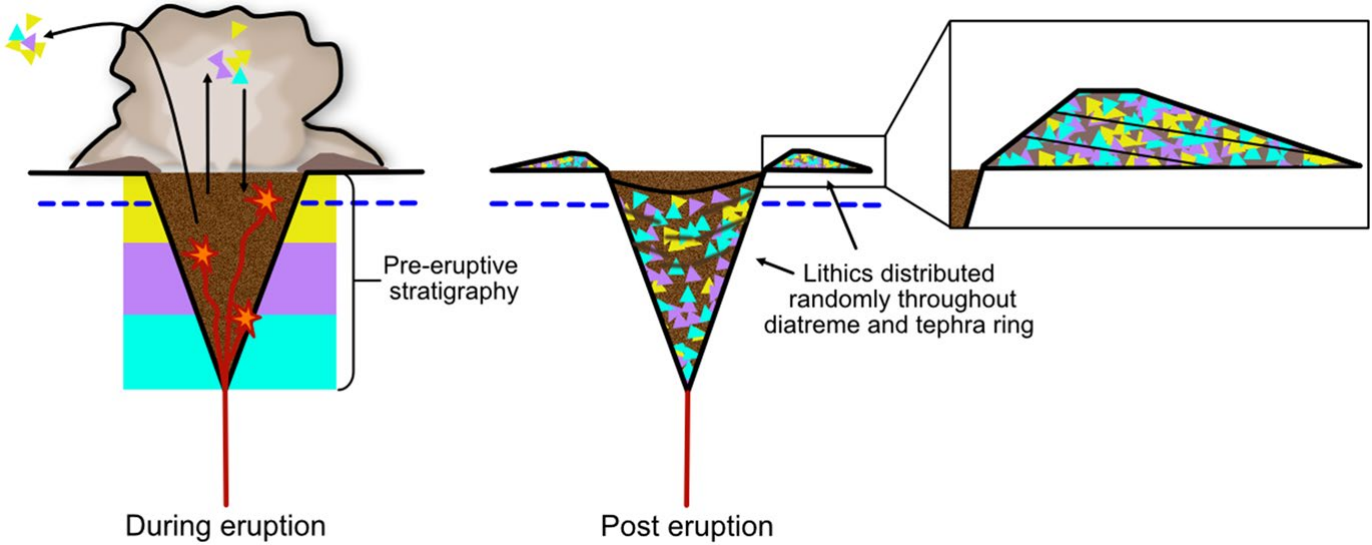
Lithic fragment behaviour expected in a revised phreatomagmatic model maar-diatreme eruption (proposed by Valentine and White^14^). Country rock and earlier-formed dikes are brecciated and then mixed within vertical jets of debris taking place within the diatreme, bolstered by subsidence of fallen pyroclasts and wall rock falling around the edges of the debris jets; these subsided products and debris jets result in variably mixed diatreme infill.

In order to address questions raised by the Lorenz model, a newer conceptual model for diatreme development by discrete explosions has been offered by Valentine and White^16^, which specifically addresses how phreatomagmatic explosions can drive the system. In this model, as magma rises up via a dike, the MFCI explosions can occur anywhere, provided the hydrostatic pressure does not exceed the critical pressure of water^16^; explosions do not necessarily occur along a downward-progressing path (Fig. 8). This creates a “proto-diatreme”, which hosts ongoing explosions with both vertically and laterally varying loci, during which the upper extent of the proto-diatreme widens more rapidly than the bottom. In the revised phreatomagmatic model, country rock and earlier-formed dikes are brecciated and then mixed within vertical jets of debris taking place within the diatreme, bolstered by subsidence of fallen pyroclasts and wall rock falling around the edges of the debris jets^14^; these subsided products and debris jets result in variably mixed diatreme infill^16^ and tephra rings dominated by shallow-derived lithic fragments^47^, e.g., Teshim maar, Hopi Buttes Volcanic Field^3^ (Fig. 11).

Results of this study show that lithic fragment characteristics in diatreme infill of both common maar-diatremes and kimberlite maar-diatremes are indeed heterogeneous and do not show any systematic patterns, which supports the concept of debris jets mixing material from different depths within the diatreme. Tephra ring material shows a slight skewing toward shallow-derived fragments throughout all stratigraphic levels of tephra rings, which supports the idea that material from approximately 200 m depth and above is more likely to be ejected onto the tephra ring^47^ and that deep-seated lithic fragments are likely to be milled within debris jets before reaching the surface (if at all). A possibility for the common angularity of fragments reported is that they could be mixed and milled fragments that experience further recycling in debris jets and then become refragmented into angular shapes as a result of subsequent subsurface explosions^48^. Combining this “debris jet” model with depth of lithic origin should, as for fluidization models, result in some correlation of shape with depth of origin. The lack of correlation found here, then, supports the notion of repeated fragmentation to explain the angularity of most fragments.

### Conclusions and information needed from future studies

This is the first review and synthesis of published data on lithic fragments in maar-diatreme volcanoes worldwide, including kimberlites. Such data has allowed us to constrain the interpretations presented below, because lithic fragments bear witness to the sites and intensity of one or more cycles of events that extract them, transport them, and deposit them. Lithic studies are important to undertake in order to improve our knowledge of the mechanisms and dynamics of explosive diatreme eruptions and evolution of shallow volcanic conduits. Our analysis of the compiled data reveals no immediate consistent correlations among any of the characteristics of lithics or their siting within the deposits surveyed. A significant finding is that there appear to be no significant correlations between lithic fragment source depths and their depositional site in the volcanoes; however, a weak correlation is that shallow-sourced lithics are the most common lithics in tephra rings. These conclusions are tentative, for there are simply not enough data available to date to claim lack of trends with full confidence—more high-quality studies on lithic fragments at diatreme and ejecta ring sites are needed for a more robust conclusion.

There is an increasing amount of lithic data being reported, which will make future analysis more straightforward. To refine models for emplacement of lithics in kimberlite and other maar-diatreme volcanoes, we must focus on improved reporting of lithic distribution, size, abundance, shape, and depths of origin. Many smaller kimberlite bodies within kimberlite fields remain unstudied and should be investigated and compared with the larger kimberlite pipes to give a broader sense of kimberlite-forming processes. There is certainly still heterogeneity in terminology and incomplete reporting of characteristics of lithic fragments in studies of maar-diatreme deposits. All lithic fragment characteristics at maar-diatreme sites should be meticulously reported with clear explanations of terminologies used; consistency in describing attributes would vastly help future analysis by allowing effective collation and comparison of data. Future work should incorporate experimentally reproducing explosions and jets variably inferred to take place in diatremes, in order to observe and determine the ways in which large particles are transported upwards in a diatreme-like granular system. Numerical modelling studies (e.g.^49^ in future work can provide another valuable dimension in analysis of lithic populations. This will aid interpretation of the results from this data compilation and analysis of lithic transport (e.g.^50^).

## Supplementary Information

Supplementary Information.

## Data Availability

All data generated or analysed during this study are included in this published article (and its Supplementary Information files).
