# Supplementary material for: A compilation and characterisation of lithics in kimberlite and common maar-diatremes and tephra ring deposits
Source: Sci Rep. 2021 Dec 14;11:24012. doi: 10.1038/s41598-021-03307-7 (PMC8671588; doi:10.1038/s41598-021-03307-7)
Supplement: Supplementary file 1 — Supplementary Information. [file 41598_2021_3307_MOESM1_ESM.docx]

Supplementary Table S1. Both common and kimberlite diatreme infill data.

| VOLCANO NAME | LITHIC FRAGMENT SIZES (mm) | Phi | SHAPE | POSITION | MIN depth (m) | MAX depth (m) | Type |
| --- | --- | --- | --- | --- | --- | --- | --- |
| RZ HBVF (White, 1991 [1]) | 4096 | -12 | 0 | Lower | 0 | 200 | Maar-diatreme |
| LD HBVF (White, 1991 [1]) | 2000 | -11 | 0 | Lower | 0 | 200 | Maar-diatreme |
| UD HBVF (White, 1991 [1]) | 4096 | -12 | 0 | Upper | 0 | 150 | Maar-diatreme |
| Missouri River Breaks Volcanic Field (Delpit et al., 2014 [2]) | 10000 | -13.3 | 2.5 | Middle | 0 | 200 | Maar-diatreme |
| Standing Rocks West, HBVF (Lefebvre et al., 2013 [3]) | 2000 | -11 | 1 | Lower | 75 | 400 | Maar-diatreme |
| U Yangpori Diatreme (Son et al., 2012 [4]) | 640 | -9.3 | 1 | Upper | 200 | 300 | Maar-diatreme |
| L Yangpori Diatreme (Son et al., 2012 [4]) | 640 | -9.3 | 0 | Lower | 0 | 200 | Maar-diatreme |
| Coombs Hill (Ross and White, 2012 [5]) | 64 | -6 | 1.5 | Middle | 500 | 500 | Maar-diatreme |
| Black Rock Butte, Waipiata (Németh, 2001 [6]) | 2500 | -11.3 | 0 | Lower | 500 | 500 | Maar-diatreme |
| Mount Rawdon (Brooker and Jaireth, 1995 [7]) | 640 | -9.3 | 2 | Lower | 200 | 200 | Maar-diatreme |
| Lower Suoana Maar Diatreme (Geshi et al., 2011 [8]) | 4096 | -12 | 1 | Lower | 220 | 220 | Maar-diatreme |
| Upper Suoana Maar Diatreme (Geshi et al., 2011 [8]) | 1000 | -10 | 1 | Upper | 220 | 220 | Maar-diatreme |
| SW Sardinia (Mundula et al., 2013 [9]) | 500 | -9 | 1 | Lower | 400 | 500 | Maar-diatreme |
| Chubut (Németh et al., 2007 [10]) | 256 | -8 | 1 | Lower | 200 | 200 | Maar-diatreme |
| The Ngatatura Diatreme (Heming, 1980 [11]) | 200 | -7.6 | 0 | Lower | 0 | 200 | Maar-diatreme |
| Aguas Emendadas Diatreme (Junquiera-Brod et al., 2004 [12]) | 1000 | -10 | 1 | Upper | 0 | 900 | Maar-diatreme |
| Neuzinha Diatreme (Junquiera-Brod et al., 2004 [12]) | 1000 | -10 | 3 | Middle | 0 | 900 | Maar-diatreme |
| Maegok Diatreme (Kwon and Sohn, 2008 [13]) | 100 | -6.6 | 3.5 | Lower | 200 | 300 | Maar-diatreme |
| Santa Cruz (Vikre et al., 2014 [14]) | 1000 | -10 | 0 | Upper | 670 | 670 | Maar-diatreme |
| Fekete-Hegy a (Auer et al., 2007 [15]) | 80 | -6.3 | 1 | Upper | 1000 | 1000 | Maar-diatreme |
| Fekete-Hegy b (Auer et al., 2007 [15]) | 100 | -6.6 | 1 | Upper | 1000 | 1000 | Maar-diatreme |
| Black Peak (Barrington and Kerr, 1961 [16]) | 120 | -6.9 | 3 | Lower | 850 | 850 | Maar-diatreme |
| Cathedral Cliff (Belanger and Ross, 2018 [17]) | - | - | 3 | Upper | - | - | Maar-diatreme |
| Cathedral Cliff (Belanger and Ross, 2018 [17]) | 20000 | -14.3 | 3 | Lower | - | - | Maar-diatreme |
| Hillier Bay (Brenna and Gee, 2014 [18]) | 100 | -6.6 | 1 | Lower | 200 | 500 | Maar-diatreme |
| Chah Gaz diatreme (Foerster and Jafarzadeh, 1994 [19]) | - | - | 0 | Upper | 2000 | 2000 | Maar-diatreme |
| Missouri River Breaks Volcanic Field (Hearn, 1968 [20]) | 640 | -9.3 | 2.5 | Lower | 1500 | 1500 | Maar-diatreme |
| Nuevo Entredicho Deposit Diatreme (Jébrak et al., 2001 [21]) | 200 | -7.6 | 0 | - | 0 | 100 | Maar-diatreme |
| Twin Peaks, HBVF (Latutrie and Ross, 2020 [22]) | 640 | -9.3 | 1.5 | Upper | 0 | 170 | Maar-diatreme |
| Upper Mount Dingley Diatreme (Norford and Cecile, 1994 [23]) | 4096 | -12 | 2 | Upper | 600 | 1000 | Maar-diatreme |
| Lower Mount Dingley Diatreme (Norford and Cecile, 1994 [23]) | 1000 | -10 | 0 | Lower | 600 | 1000 | Maar-diatreme |
| Jagged Rocks, HBVF (Re et al., 2016 [24]) | 500 | -9 | 0 | Lower | 380 | 380 | Maar-diatreme |
| Upper Mardoux Structure (Valentine and Van Wyck DeVries, 2014 [25]) | 256 | -8 | 2.5 | Upper | 0 | 200 | Maar-diatreme |
| Lower Mardoux Structure (Valentine and Van Wyck DeVries, 2014 [25]) | 640 | -9.3 | 4 | Upper | 0 | 200 | Maar-diatreme |
| Standing Rocks East, HBVF (White et al., 2013 [26]) | 640 | -9.3 | 0 | Lower | 0 | 500 | Maar-diatreme |
| Kisselworth Diatreme (Lutz et al., 2013 [27]) | 50 | -5.6 | 1 | Upper | - | - | Maar-diatreme |
| Windy Point Diatreme (Hayward, 1977 [28]) | 400 | -8.6 | 0 | Lower | 200 | 500 | Maar-diatreme |
| LFA3 Elie Ness (Gernon et al., 2013 [29]) | 640 | -9.3 | 1 | - | - | - | Maar-diatreme |
| LFA1a Limerick (Gernon et al., 2015 [30]) | 7 | -2.8 | 2.5 | - | 0 | 130 | Maar-diatreme |
| LFA1b Limerick (Gernon et al., 2015 [30]) | 16 | -4 | 2.5 | - | 0 | 130 | Maar-diatreme |
| LFA2 Limerick (Gernon et al., 2015 [30]) | 39 | -5.3 | 4 | - | 0 | 80 | Maar-diatreme |
| Ebersbrunn Diatreme (Schmidt et al., 2013 [31]) | 30 | -4.9 | 1 | Lower | - | - | Maar-diatreme |
| Tumpangpitu Diatreme (Harrison et al., 2018 [32]) | 10000 | -13.3 | 3 | Upper | - | - | Maar-diatreme |
| Upper Round Butte (Latutrie and Ross, 2019 [33]) | 10000 | -13.3 | 0 | Upper | 0 | 100 | Maar-diatreme |
| Lower Round Butte (Latutrie and Ross, 2019 [33]) | - | - | 0 | Lower | 0 | 440 | Maar-diatreme |
| Szigliget Unit 1 (Németh et al., 2000 [34]) | 640 | -9.3 | 2 | Lower | 200 | 200 | Maar-diatreme |
| La Crosa de Sant Delmai Maar (Bolós et al., 2012 [35]) | 700 | -9.5 | 1.5 | Upper | 35 | 200 | Maar-diatreme |
| MMU 147 Kimberlite, Fort a la Corne (Lefebvre and Kurszlaukis, 2008 [36]) | 22000 | -14.4 | 0 | - | 200 | 500 | Kimberlite |
| Upper Pigeon Kimberlite (Crawford et al., 2009 [37]) | 40 | -5.3 | 4 | Upper | 200 | 200 | Kimberlite |
| Lower Pigeon Kimberlite (Crawford et al., 2009 [37]) | - | - | 1 | Lower | 200 | 200 | Kimberlite |
| Renard 65a (Gaudet et al., 2018 [38]) | 15000 | -13.9 | 1 | Lower | 565 | 565 | Kimberlite |
| Renard 65b (Gaudet et al., 2018 [38]) | 300 | -8.2 | 2.5 | Lower | 565 | 565 | Kimberlite |
| Renard 65d (Gaudet et al., 2018 [38]) | 450 | -8.8 | 2.5 | Lower | 565 | 565 | Kimberlite |
| K2 West CR megablocks (Brown et al., 2009 [39]) | 20000 | -14.3 | 2.5 | Upper | 900 | 900 | Kimberlite |
| K2 West CR Breccia (Brown et al., 2009 [39]) | 64 | -6 | 1 | Upper | 900 | 900 | Kimberlite |
| K2 West Kim Breccia (Brown et al., 2009 [39]) | 640 | -9.3 | 2.5 | Upper | 900 | 900 | Kimberlite |
| K2 East MVK (Brown et al., 2009 [39]) | - | - | 1 | Lower | 900 | 900 | Kimberlite |
| K2 West CK (Brown et al., 2008 [39]) | 300 | -8.2 | 0 | Lower | - | - | Kimberlite |
| BK9 - DVK (ns) (Buse et al., 2011 [40]) | 260 | -8 | 4 | Lower | 0 | 200 | Kimberlite |
| BK9 - DVK (c) (Buse et al., 2011 [40]) | 200 | -7.6 | 1 | Upper | 0 | 200 | Kimberlite |
| Venetia K1 (Gernon et al., 2009a [41]) | 120 | -6.9 | 4 | Lower | 0 | 200 | Kimberlite |
| U1 Orapa South Pipe (Gernon et al., 2009b [42]) | 1024 | -10 | 1.5 | Lower | 0 | 200 | Kimberlite |
| U2 Orapa South Pipe (Gernon et al., 2009b [42]) | 1000 | -10 | 1.5 | Lower | 0 | 200 | Kimberlite |
| U3 Orapa South Pipe (Gernon et al., 2009b [42]) | 64 | -6 | 1 | Upper | 0 | 200 | Kimberlite |
| U4 Orapa South Pipe (Gernon et al., 2009b [42]) | 2900 | -11.5 | 1 | Upper | 0 | 200 | Kimberlite |
| U6 Orapa South Pipe (Gernon et al., 2009b [42]) | 1330 | -10.4 | 2 | Upper | 0 | 200 | Kimberlite |
| U7 Orapa South Pipe (Gernon et al., 2009b [42]) | 256 | -8 | 2.5 | Upper | 0 | 200 | Kimberlite |
| Cane Valley Diatreme (McGetchin and Nikhanj, 1973 [43]) | 4096 | -12 | 0 | Upper | - | - | Kimberlite |
| SVK A154N Pipe, Diavik (Moss et al., 2008 [44]) | 200 | -7.6 | 1 | Upper | - | - | Kimberlite |
| MVK Venetia K1 (Walters et al., 2006 [45]) | 30 | -4.9 | 1 | Lower | - | - | Kimberlite |
| CRB Venetia K2 (Walters et al., 2006 [45]) | 3000 | -11.6 | 1 | Upper | - | - | Kimberlite |
| F1 Fox Kimberlite (Porritt and Cas, 2009 [46]) | 1024 | -10 | 1 | Lower | - | - | Kimberlite |
| F2 Fox Kimberlite (Porritt and Cas, 2009 [46]) | 1024 | -10 | 1 | Upper | - | - | Kimberlite |
| Basalt Orapa North Pipe (Gernon et al., 2008 [47]) | 100 | -6.6 | 1 | Lower | 0 | 200 | Kimberlite |
| Basement Orapa North Pipe (Gernon et al., 2008 [47]) | 100 | -6.6 | 3 | Lower | 200 | 500 | Kimberlite |
| CRB K08 Pipe (Barnett et al., 2011 [48]) | 10000 | -13.3 | 0 | Upper | - | - | Kimberlite |
| HK K08 Pipe (Barnett et al., 2011 [48]) | 1200 | -10.2 | 0 | Lower | - | - | Kimberlite |
| TK Tuzo Pipe (Seghedi et al., 2009 [49]) | 1000 | -10 | 0 | Lower | - | - | Kimberlite |
| LFA1 MVK Jwaneng Centre Pipe (Brown et al., 2008 [50]) | 40 | -5.3 | 0 | Lower | - | - | Kimberlite |
| LFA1 BVK Jwaneng Centre Pipe (Brown et al., 2008 [50]) | 40 | -5.3 | 0 | Lower | - | - | Kimberlite |
| LFA1 Bs Jwaneng Centre Pipe (Brown et al., 2008 [50]) | 100 | -6.6 | 1 | Lower | - | - | Kimberlite |
| LFA2 MVKm Jwaneng Centre Pipe (Brown et al., 2008 [50]) | - | - | 2.5 | Lower | - | - | Kimberlite |
| LFA2 BVKBm Jwaneng Centre Pipe (Brown et al., 2008 [50]) | 1024 | -10 | 0 | Lower | - | - | Kimberlite |
| LFA3 BLvs Jwaneng Centre Pipe (Brown et al., 2008 [50]) | 10000 | -13.3 | 0 | Upper | - | - | Kimberlite |
| 118 FALC (Scott Smith, 2008 [51]) | 140 | -7.1 | 0 | Upper | 0 | 90 | Kimberlite |
| 118 FALC (Scott Smith, 2008 [51]) | 30 | -4.9 | 0 | Upper | 570 | 570 | Kimberlite |
| Green Knobs, NM, Colorado Plateau (Smith and Levy, 1975 [52]) | 1000 | -10 | 3 | - | - | - | Kimberlite |

Supplementary Table S2. Both common and kimberlite tephra ring data.

| VOLCANO NAME | LITHIC FRAGMENT SIZES (mm) | Phi | SHAPE | POSITION | MIN depth (m) | MAX depth (m) | Type |
| --- | --- | --- | --- | --- | --- | --- | --- |
| Motukorea Volcano (Agustín-Flores et al., 2015 [53]) | 40 | -5.3 | 3 | Lower | 0 | 300 | Maar tephra |
| HBVF – TBm (Graettinger and Valentine, 2017 [54]) | 600 | -9.2 | 3 | Lower | 0 | 50 | Maar tephra |
| HBVF – Atx (Graettinger and Valentine, 2017 [54]) | 40 | -5.3 | 2.5 | Middle | 0 | 390 | Maar tephra |
| HBVF – TBg (Graettinger and Valentine, 2017 [54]) | 300 | -8.2 | 2.5 | Middle | 170 | 390 | Maar tephra |
| HBFV – LTw (Graettinger and Valentine, 2017 [54]) | 40 | -5.3 | 2.5 | Upper | - | - | Maar tephra |
| Pula Maar Early (Németh et al., 2008 [55]) | 64 | -6 | 1 | Lower | 0 | 300 | Maar tephra |
| Pula Maar Upper (Németh et al., 2008 [55]) | 40 | -5.3 | 1 | Middle | 300 | 300 | Maar tephra |
| Stracciacappa Maar (Valentine et al., 2015 [56]) | 700 | -9.5 | 0 | Lower | 450 | 650 | Maar tephra |
| Finca la Nava Maar (Lierenfeld and Mattson, 2015 [57]) | 400 | -8.6 | 1 | Lower | 0 | 400 | Maar tephra |
| Teshim, HBVF (Lefebvre et al., 2013 [3]) | - | - | 0 | Lower | 0 | 50 | Maar tephra |
| Teshim Maar (White, 1991 [1]) | 200 | -7.6 | 2.5 | Upper | 50 | 150 | Maar tephra |
| Dry Lake Maar, SFVF Lower (Valentine, 2012 [58]) | 100 | -6.6 | 2.5 | Lower | 50 | 50 | Maar tephra |
| Dry Lake Maar, SFVF Middle (Valentine, 2012 [59]) | 640 | -9.3 | 0 | Middle | 200 | 500 | Maar tephra |
| Dry Lake Maar, SFVF Upper (Valentine, 2012 [58]) | 480 | -8.9 | 0 | Upper | 50 | 50 | Maar tephra |
| Rattlesnake Crater (Valentine, 2012 [58]) | 210 | -7.7 | 0 | Lower | 0 | 140 | Maar tephra |
| Martignano Composite Maar (Sottili et al., 2011 [59]) | 500 | -9 | 1 | Lower | 400 | 600 | Maar tephra |
| East Maar, Pali Aike Volcanic Field (Ross et al., 2011 [60]) | 50 | -5.6 | 1.5 | Lower | 0 | 200 | Maar tephra |
| Cora Maar (Gençalioğlu-Kuşcu et al., 2007 [61]) | 1300 | -10.3 | 0 | Lower | 0 | 200 | Maar tephra |
| Barombi Mbo Maaar U1 (Tchamabé et al., 2013 [62]; 2014 [63]; 2015 [64]) | 40 | -5.3 | 0 | Lower | 200 | 700 | Maar tephra |
| Barombi Mbo Maar U2 (Tchamabé et al., 2013 [62]; 2014 [63]; 2015 [64]) | 100 | -6.6 | 1 | Middle | 200 | 700 | Maar tephra |
| Barombi Mbo Maaar U3 (Tchamabé et al., 2013 [62]; 2014 [63]; 2015 [64]) | 500 | -9 | 0 | Upper | 200 | 700 | Maar tephra |
| Tiscapa Maar (Freundt et al., 2010 [65]) | 40 | -5.3 | 0 | Lower | - | - | Maar tephra |
| South Alkali Butte, Lucero (Valentine and Groves, 1996 [66]) | 2200 | -11.1 | 0 | Lower | 500 | 700 | Maar tephra |
| Split Butte, Snake River Plain Idaho (Womer et al., 1980 [67]) | 1250 | -10.3 | 1 | Lower | 200 | 200 | Maar tephra |
| Basalt Fragments Bea's Crater - Lunar Crater Volcanic Field (Amin and Valentine, 2017 [68]) | 500 | -9 | 1 | Upper | 0 | 250 | Maar tephra |
| Rhyolite Fragments - Bea's Crater - Lunar Crater Volcanic Field (Amin and Valentine, 2017 [68]) | 500 | -9 | 1 | Upper | 150 | 1800 | Maar tephra |
| Bea's Crater - Lunar Crater Volcanic Field (Amin and Valentine, 2017 [68]) | 640 | -9.3 | 1.5 | Lower | 0 | 250 | Maar tephra |
| Narkoy Maar (Gevrek and Kazanci, 2000 [69]) | 800 | -9.6 | 1.5 | Upper | - | - | Maar tephra |
| Suoana Maar (Geshi et al., 2011 [8]) | 2000 | -11 | 1 | Upper | 220 | 220 | Maar tephra |
| Alchichica Maar Lower (Tchamabé et al., 2020 [70]) | 640 | -9.3 | 1.5 | Lower | - | - | Maar tephra |
| Alchichica Maar Middle (Tchamabé et al., 2020 [70]) | 1024 | -10 | 2 | Middle | 200 | 500 | Maar tephra |
| Alchichica Maar Upper (Tchamabé et al., 2020 [70]) | 40 | -5.3 | 1 | Upper | 200 | 500 | Maar tephra |
| Solfatara Maar-Diatreme (Isaia et al., 2015 [71]) | 1000 | -10 | 4 | Upper | 200 | 500 | Maar tephra |
| Lake Purrumbete Maar Upper (Jordan et al., 2013 [72]) | 40 | -5.3 | 0 | Upper | 0 | 250 | Maar tephra |
| Lake Purrumbete Maar Lower (van den Hove et al., 2015 [73]) | 120 | -6.9 | 2.5 | Lower | 240 | 240 | Maar tephra |
| PH1 Tihany Maar (Németh et al., 2001 [74]) | 50 | -5.6 | 2.5 | Lower | 200 | 400 | Maar tephra |
| PH2 Tihany Maar (Németh et al., 2001 [74]) | 250 | -8 | 0 | Middle | 200 | 800 | Maar tephra |
| PH3 Tihany Maar (Németh et al., 2001 [74]) | 50 | -5.6 | 0 | Upper | 200 | 600 | Maar tephra |
| Debunscha Maar (Ngwa et al., 2010 [75]) | 500 | -9 | 1.5 | Lower | - | - | Maar tephra |
| Camp del Ninots Maar-Diatreme (Oms et al., 2015 [76]) | 500 | -9 | 1.5 | Lower | 300 | 300 | Maar tephra |
| Rotomahana (Pittari et al., 2016 [77]) | 100 | -6.6 | 1.5 | Lower | 0 | 200 | Maar tephra |
| Los Marteles Caldera (Sarrionandia et al., 2015 [78]) | 600 | -9.2 | 1.5 | Lower | - | - | Maar tephra |
| Duraznero (White and Schmincke, 1999 [79]) | 640 | -9.3 | 0 | Lower | 0 | 200 | Maar tephra |
| Hoyo Negro Early (White and Schmincke, 1999 [79]) | 300 | -8.2 | 0 | Lower | 0 | 200 | Maar tephra |
| Hoyo Negro Later (White and Schmincke, 1999 [79]) | 1000 | -10 | 0 | Middle | 0 | 200 | Maar tephra |
| Saefell A/B (Mattsson et al., 2005 [80]) | 43 | -5.4 | 1 | Lower | 0 | 170 | Maar tephra |
| Saefell C/D (Mattsson et al., 2005 [80]) | 52 | -5.7 | 0 | Middle | 200 | 400 | Maar tephra |
| Saefell E/T (Mattsson et al., 2005 [80]) | 73 | -6.2 | 0 | Upper | 820 | 820 | Maar tephra |
| Southern Amphitheater (Fierstein and Hildreth, 2017 [81]) | 1300 | -10.3 | 4 | Lower | 200 | 500 | Maar tephra |
| Middle Amphitheater (Fierstein and Hildreth, 2017 [81]) | 2000 | -11 | 1 | Middle | 200 | 500 | Maar tephra |
| Northern Amphitheater (Fierstein and Hildreth, 2017 [81]) | 60 | -5.9 | 4 | Upper | - | - | Maar tephra |
| Crater P (Fierstein and Hildreth, 2017 [81]) | 900 | -9.8 | 0 | Upper | 200 | 500 | Maar tephra |
| The Crater, Waipiata (Németh, 2001 [6]) | 64 | -6 | 0 | Lower | 0 | 300 | Maar tephra |
| Asososca Tephra (Pardo et al., 2009 [82]) | 640 | -9.3 | 1 | Lower | 0 | 200 | Maar tephra |
| NNT-A (Pardo et al.,2008 [83]; 2009 [82]) | 1000 | -10 | 0 | Lower | 274 | 274 | Maar tephra |
| NNT-B (Pardo et al., 2008 [83]; 2009 [82]) | 200 | -7.6 | 2 | Middle | 274 | 274 | Maar tephra |
| NNT-C (Pardo et al., 2008 [83]; 2009 [82]) | 640 | -9.3 | 2 | Upper | 274 | 274 | Maar tephra |
| Unit III, Tecuitlapa Maar (Ort and Carrasco-Núñez, 2009 [84]) | 1500 | -10.6 | 0 | Upper | 0 | 100 | Maar tephra |
| Lower East Maar, Ukinrek (Self et al., 1980 [85]) | 38 | -5.2 | 0 | Lower | - | - | Maar tephra |
| Upper East Maar, Ukinrek (Self et al., 1980 [85]) | 28 | -4.8 | 2 | Upper | - | - | Maar tephra |
| Lunar Crater (Valentine et al., 2011 [86]) | 640 | -9.3 | 0 | Upper | 0 | 180 | Maar tephra |
| Szigliget Unit 2 (Németh et al., 2000 [34]) | 100 | -6.6 | 0 | Lower | 200 | 200 | Maar tephra |
| Szigliget Unit 3 (Németh et al., 2000 [34]) | 40 | -5.3 | 0 | Upper | 0 | 200 | Maar tephra |
| Lower Joya Honda (Aranda-Gomez and Luhr, 1996 [87]) | 200 | -7.6 | 3 | Lower | 200 | 300 | Maar tephra |
| Upper Joya Honda (Aranda-Gomez and Luhr, 1996 [87]) | 150 | -7.2 | 3 | Upper | 200 | 300 | Maar tephra |
| Puig d’Àdri Unit 1 (Pedrazzi et al., 2016 [88]) | 40 | -5.3 | 0 | Lower | 0 | 350 | Maar tephra |
| Puig d’Àdri Unit 3 (Pedrazzi et al., 2016 [88]) | 10 | -3.3 | 1 | Middle | 0 | 350 | Maar tephra |
| Puig d’Àdri Unit 4 (Pedrazzi et al., 2016 [88]) | 50 | -5.6 | 0 | Upper | 0 | 350 | Maar tephra |
| EJF, Orion South/Star (Harvey et al., 2009 [89]; Zonneveld et al., 2004 [90]; Scott Smith, 2008 [51]) | 70 | -6.1 | 2 | Lower | 170 | 650 | Kimberlite tephra |
| MJF, Orion South/Star (Harvey et al., 2009 [89]; Zonneveld et al., 2004 [90]; Scott Smith, 2008 [51]) | - | - | 2.5 | Middle | 0 | 90 | Kimberlite tephra |
| LJF, Orion South/Star (Harvey et al., 2009 [89]; Zonneveld et al., 2004 [90]; Scott Smith, 2008 [51]) | 75 | -6.2 | 2.5 | Upper | 0 | 90 | Kimberlite tephra |
| 169 Smeaton (Leckie et al., 1997 [91]) | 30 | -4.9 | 0 | Lower | 170 | 650 | Kimberlite tephra |
| Igwisi NE Volcano (Brown et al., 2012 [92]) | 70 | -6.1 | 2 | Lower | - | - | Kimberlite tephra |

**
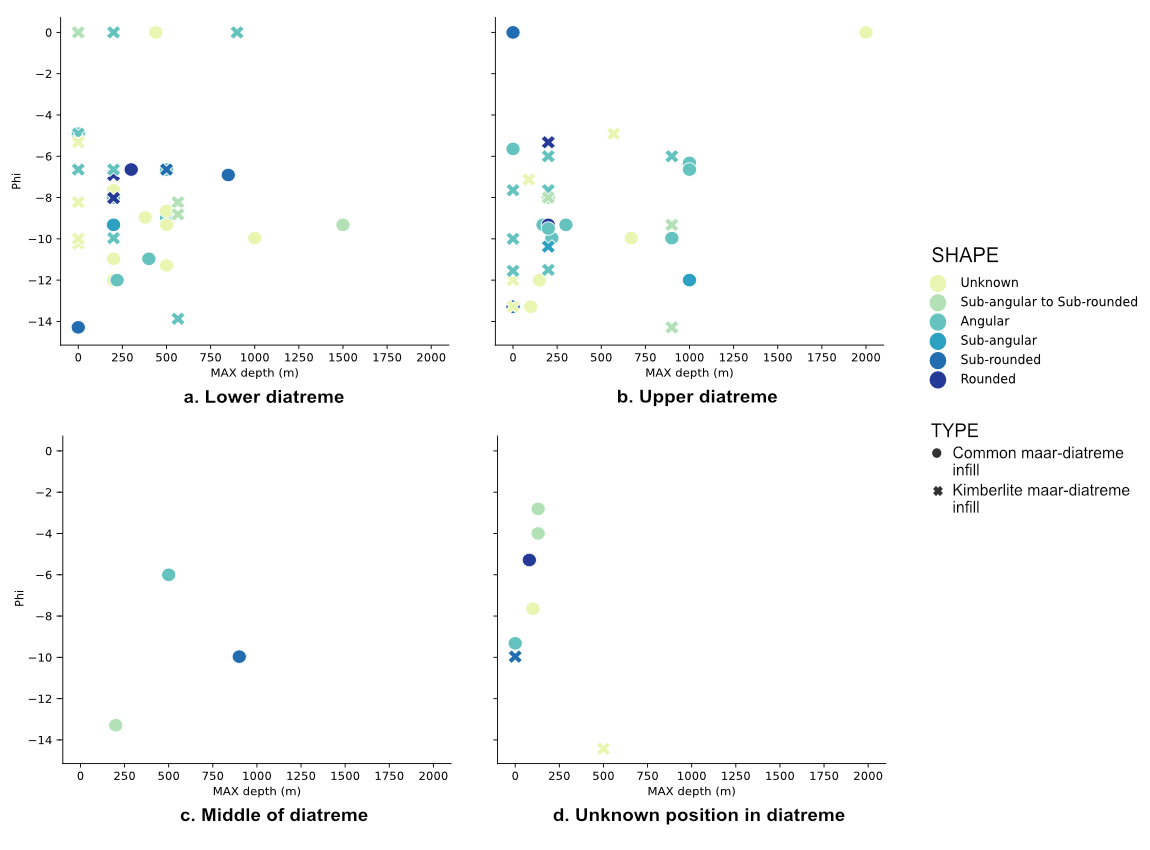
**

Supplementary Figure 1. Each part of the diatreme infill sequence plotted to show final position in diatreme, maximum original depth, phi, and shape for lithics in both common maar-diatreme infill and kimberlite maar-diatreme infill deposits. a = lower; b = upper; c = middle; d = unknown diatreme area. Where information for size (phi) or original depth was not given, these are plotted as “0”.


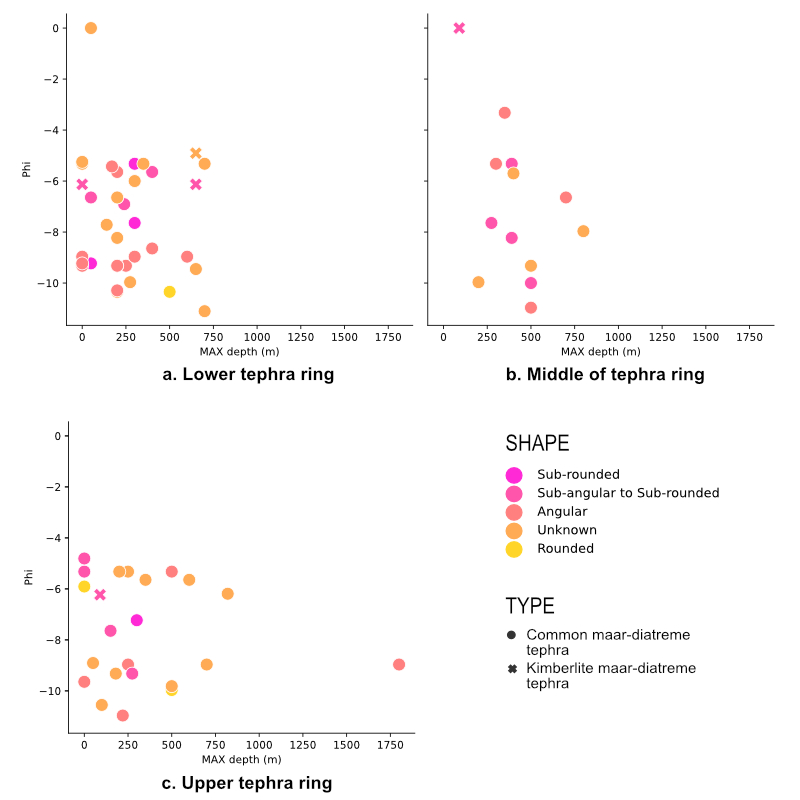


Supplementary Figure 2. Each part of the tephra ring sequence plotted to show final position in tephra ring, maximum original depth, phi, and shape for lithics in both common maar-diatreme tephra ring and kimberlite maar-diatreme tephra ring deposits. a = lower; b = middle; c = upper tephra ring. Where information for size (phi) or original depth was not given, these are plotted as “0”.

**References**

1. White, J.D.L. Maar-diatreme phreatomagmatism at Hopi Buttes, Navajo Nation (Arizona), USA. *Bull Volcanol*. **53,** 239–258 (1991).

2. Delpit, S., Ross, P.-S. & Hearn, B.C. Deep-bedded ultramafic diatremes in the Missouri River Breaks volcanic field, Montana, USA: 1 km of syn-eruptive subsidence. *Bull. Volcanol.* **76,** 832 (2014).

3. Lefebvre, N.S., White, J.D.L. & Kjarsgaard, B.A. Unbedded diatreme deposits reveal maar-diatreme-forming eruptive processes: Standing Rocks West, Hopi Buttes, Navajo Nation, USA. *Bull Volcanol.* **75,** 739 (2013).

4. Son, M. *et al.* Tectonically controlled vent migration during maar–diatreme formation: An example from a Miocene half-graben basin in SE Korea. *J Volcanol Geotherm Res.* **223–224,** 29–46 (2012).

5. Ross, P.-S. & White, J.D.L. Quantification of vesicle characteristics in some diatreme-filling deposits, and the explosivity levels of magma–water interactions within diatremes. *J Volcanol Geotherm Res.* **245–246,** 55–67 (2012).

6. Németh, K. Long-term erosion-rate calculation from the Waipiata Volcanic Field (New Zealand) based on erosion remnants of scoria cones, tuff rings and maars. *Géomorphologie*. **2,** 137–152 (2001).

7. Brooker, M. & Jaireth, S. Mount Rawdon, Southeast Queensland, Australia; a diatreme-hosted gold-silver deposit. *Econ Geol.* **90,** 1799–1817 (1995).

8. Geshi, N., Németh, K. & Oikawa, T. Growth of phreatomagmatic explosion craters: a model inferred from Suoana crater in Miyakejima Volcano, Japan. *J Volcanol Geotherm Res.* **201,** 30–38 (2011).

9. Mundula, F., Cioni, R., Funedda, A. & Leone, F. Lithofacies characteristics of diatreme deposits: examples from a basaltic volcanic field of SW Sardinia (Italy). *J Volcanol Geotherm Res.* **255,** 1–14 (2013).

10. Németh, K., Martin, U., Haller, M.J. & Alric, V.I. Cenozoic diatreme field in Chubut (Argentina) as evidence of phreatomagmatic volcanism accompanied with extensive Patagonian plateau basalt volcanism? *Episodes.* **30,** 217–223 (2007).

11. Heming, R.F. The Ngatatura diatreme. *NZJGG.* **23,** 569–573 (1980).

12. Junqueira-Brod, T.C., Brod, J.A., Gaspar, J.C. & Jost, H. Kamafugitic diatremes: facies characterisation and genesis—examples from the Goiás Alkaline Province, Brazil. *Lithos.* **76,** 261–282 (2004).

13. Kwon, C.W. & Sohn, Y.K. Tephra-filled volcanic neck (diatreme) of a mafic tuff ring at Maegok, Miocene Eoil Basin, SE Korea. *Geosci J.* **12,** 317–329 (2008).

14. Vikre, P.G., Graybeal, F.T. & Koutz, F.R. Concealed basalt-matrix diatremes with Cu-Au-Ag-(Mo)-mineralized xenoliths, Santa Cruz porphyry Cu-(Mo) system, Pinal County, Arizona. *Econ Geol.* **109,** 1271–1289 (2014).

15. Auer, A., Martin, U. & Németh, K. The Fekete-hegy (Balaton Highland Hungary) “soft-substrate” and “hard-substrate” maar volcanoes in an aligned volcanic complex—implications for vent geometry, subsurface stratigraphy and the palaeoenvironmental setting. *J Volcanol Geotherm Res.* **159,** 225–245 (2007).

16. Barrington, J. & Kerr, P.F. Breccia Pipe near Cameron, Arizona. *Geol Soc Am Bull.* **72,** 1661–1674 (1961).

17. Bélanger, C. & Ross, P.-S. Origin of nonbedded pyroclastic rocks in the Cathedral Cliff diatreme, Navajo volcanic field, New Mexico. *Bull Volcanol*. **80,** 1–18 (2018).

18. Brenna, M. & Gee, M.A.M. Dyke-diatreme transition in monogenetic volcanoes: insights from the Hillier Bay volcanic complex, Western Australia. *Bull Volcanol.* **76,** 1–13 (2014).

19. Foerster, H. & Jafarzadeh, A. The Bafq mining district in central Iran; a highly mineralized Infracambrian volcanic field. *Econ Geol.* **89,** 1697–1721 (1994).

20. Hearn, B.C. Diatremes with kimberlitic affinities in North-Central Montana. *Science*. **159,** 622–625 (1968).

21. Jébrak, M., Higueras, P.L., Marcoux, É. & Lorenzo, S. Geology and geochemistry of high-grade, volcanic rock-hosted, mercury mineralisation in the Nuevo Entredicho deposit, Almadén district, Spain. *Miner Depos***. 37,** 421–432 (2002).

22. Latutrie, B. & Ross, P.S. Phreatomagmatic vs magmatic eruptive styles in maar-diatremes: a case study at Twin Peaks, Hopi Buttes volcanic field, Navajo Nation, Arizona. *Bull Volcanol.* **82(3),** 1–25 (2020).

23. Norford, B.S. & Cecile, M.P. Ordovician emplacement of the Mount Dingley Diatreme, Western Ranges of the Rocky Mountains, southeastern British Columbia. *Can. J. Earth Sci.* **31,** 1491–1500 (1994).

24. Re, G., White, J., Muirhead, J.D. & Ort, M.H. Subterranean fragmentation of magma during conduit initiation and evolution in the shallow plumbing system of the small-volume Jagged Rocks volcanoes (Hopi Buttes Volcanic Field, Arizona, USA). *Bull Volcanol.* **78,** 1–20 (2016).

25. Valentine, G.A. & van Wyk de Vries, B. Unconventional maar diatreme and associated intrusions in the soft sediment-hosted Mardoux structure (Gergovie, France). *Bull Volcanol*. **76,** 807 (2014).

26. White, J., Lefebvre, N. & Kjarsgaard, B. Bombs, welded spatter, rockfall and cross-cutting breccia enclosed in avalanche deposits 300 m deep in a debris-filled vent (diatreme), Hopi Buttes, Arizona. *EGU General Assembly Conference Abstracts.* 2013–8558 (2013).

27. Lutz, H., Lorenz, V., Engel, T., Häfner, F. & Haneke, J. Paleogene phreatomagmatic volcanism on the western main fault of the northern Upper Rhine Graben (Kisselwörth diatreme and Nierstein–Astheim Volcanic System, Germany). *Bull Volcanol.* **75,** 741 (2013).

28. Hayward, B.W. Miocene volcanic centres of the Waitakere Ranges, North Auckland, New Zealand. *J R Soc NZ.* **7,** 123–141 (1977).

29. Gernon, T.M., Upton, B.G.J. & Hincks, T.K. Eruptive history of an alkali basaltic diatreme from Elie Ness, Fife, Scotland. *Bull Volcanol.* **75**, 704 (2013).

30. Gernon, T.M., Roberts, S., Hewson, C. & Elliott, H. Basaltic maar-diatreme volcanism in the Lower carboniferous of the Limerick Basin (SW Ireland). *Bull Volcanol.* **77,** 1–22 (2015).

31. Schmidt, A. *et al*. Origin of magnetic anomalies in the large Ebersbrunn diatreme, W Saxony, Germany. *Bull Volcanol.* **75,** 766 (2013).

32. Harrison, R.L. *et al*. Geochronology of the Tumpangpitu porphyry Au-Cu-Mo and high-sulfidation epithermal Au-Ag-Cu deposit: evidence for pre- and postmineralization diatremes in the Tujuh Bukit district, Southeast Java, Indonesia. *Econ Geol.* **113,** 163–192 (2018).

33. Latutrie, B. & Ross, P.-S. Transition zone between the upper diatreme and lower diatreme: origin and significance at Round Butte, Hopi Buttes volcanic field, Navajo Nation, Arizona. *Bull Volcanol.* **81,** 1–22 (2019).

34. Németh, K., Korbely, B. & Karatson, D. The Szigliget maar/diatreme, Bakony-Balaton Highland Volcanic Field (Hungary). *Terra Nostra* 2000/6 International Maar Conference, 375–382 (2000).

35. Bolós, X. *et al*. Investigation of the inner structure of La Crosa de Sant Dalmai maar (Catalan Volcanic Zone, Spain). *J Volcanol Geotherm Res.* **247–248,** 37–48 (2012).

36. Lefebvre, N. & Kurszlaukis, S. Contrasting eruption styles of the 147 Kimberlite, Fort à la Corne, Saskatchewan, Canada. *J Volcanol Geotherm Res.* **174,** 171–185 (2008).

37. Crawford, B., Hetman, C., Nowicki, T., Baumgartner, M. & Harrison, S. The geology and emplacement history of the Pigeon kimberlite, EKATI Diamond Mine, Northwest Territories, Canada. *Lithos*. **112,** 501–512 (2009).

38. Gaudet, M., Kopylova, M., Muntener, C., Zhuk, V. & Nathwani, C. Geology of the Renard 65 kimberlite pipe, Québec, Canada. *Miner Petrol.* **112,** 433–445 (2018).

39. Brown, R.J., Tait, M., Field, M. & Sparks, R.S.J. Geology of a complex kimberlite pipe (K2 pipe, Venetia Mine, South Africa): insights into conduit processes during explosive ultrabasic eruptions. *Bull Volcanol.* **71,** 95–112 (2009).

40. Buse, B. *et al*. Geology of the BK9 kimberlite (Damtshaa, Botswana): implications for the formation of dark volcaniclastic kimberlite. *Bull Volcanol.* **73,** 1029–1045 (2011).

41. Gernon, T. M., Gilbertson, M.A., Sparks, R.S.J. & Field, M. The role of gas-fluidisation in the formation of massive volcaniclastic kimberlite. *Lithos,* **112,** 439–451 (2009).

42. Gernon, Thomas M., Field, M. & Sparks, R.S.J. Depositional processes in a kimberlite crater: the Upper Cretaceous Orapa South Pipe (Botswana). *Sedimentology.* **56,** 623–643 (2009).

43. McGetchin, T.R., Nikhanj, Y.S. & Chodos, A.A. Carbonatite‐kimberlite relations in the Cane Valley Diatreme, San Juan County, Utah. *J Geophys Res.* **78(11),** 1854–1869 (1973).

44. Moss, S., Russell, J.K. & Andrews, G.D.M. Progressive infilling of a kimberlite pipe at Diavik, Northwest Territories, Canada: insights from volcanic facies architecture, textures, and granulometry. *J Volcanol Geotherm Res.* **174,** 103–116 (2008).

45. Walters, A.L. *et al*. The role of fluidisation in the formation of volcaniclastic kimberlite: grain size observations and experimental investigation. *J Volcanol Geotherm Res.***155,** 119–137 (2006).

46. Porritt, L.A. & Cas, R.A.F. Reconstruction of a kimberlite eruption, using an integrated volcanological, geochemical and numerical approach: a case study of the Fox Kimberlite, NWT, Canada. *J Volcanol Geotherm Res.* **179,** 241–264 (2009).

47. Gernon, T.M., Sparks, R.S.J. & Field, M. Degassing structures in volcaniclastic kimberlite: Examples from southern African kimberlite pipes. *J Volcanol Geotherm Res.* **174,** 186–194 (2008).

48. Barnett, W.P., Kurszlaukis, S., Tait, M. & Dirks, P. Kimberlite wall-rock fragmentation processes: Venetia K08 pipe development. *Bull Volcanol.* **73,** 941–958 (2011).

49. Seghedi, I., Maicher, D. & Kurszlaukis, S. Volcanology of Tuzo pipe (Gahcho Kué cluster) — root–diatreme processes re-interpreted. *Lithos.* **112,** 553–565 (2009).

50. Brown, R.J., Gernon, T., Stiefenhofer, J. & Field, M. Geological constraints on the eruption of the Jwaneng Centre kimberlite pipe, Botswana. *J Volcanol Geotherm Res.* **174,** 195–208 (2008).

51. Scott Smith, B.H. The Forte a la Corne kimberlites, Saskatchewan, Canada: geology, emplacement and economics. *J Geol Soc India.* **71,** 11–55 (2008).

52. Smith, D. & Levy, S. Petrology of the Green Knobs diatreme and implications for the upper mantle below the Colorado Plateau. *Earth Planet Sci Lett.* **29,** 107–125 (1976).

53. Agustín-Flores, J., Németh, K., Cronin, S.J., Lindsay, J.M. & Kereszturi, G. Shallow-seated explosions in the construction of the Motukorea tuff ring (Auckland, New Zealand): evidence from lithic and sedimentary characteristics. *J Volcanol Geotherm Res.* **304,** 272–286 (2015).

54. Graettinger, A.H. & Valentine, G.A. Evidence for the relative depths and energies of phreatomagmatic explosions recorded in tephra rings. *Bull Volcanol.* **79,** 1–21 (2017).

55. Németh, K., Goth, K., Martin, U., Csillag, G. & Suhr, P. Reconstructing paleoenvironment, eruption mechanism and paleomorphology of the Pliocene Pula maar, (Hungary). *J Volcanol Geotherm Res.* **177,** 441–456 (2008).

56. Valentine, G.A., Sottili, G., Palladino, D.M. & Taddeucci, J. Tephra ring interpretation in light of evolving maar–diatreme concepts: Stracciacappa maar (central Italy). *J Volcanol Geotherm Res.* **308,** 19–29 (2015).

57. Lierenfeld, M.B. & Mattsson, H.B. Geochemistry and eruptive behaviour of the Finca la Nava maar volcano (Campo de Calatrava, south-central Spain). *Int J Earth Sci*. **104,** 1795–1817 (2015).

58. Valentine, G.A. Shallow plumbing systems for small-volume basaltic volcanoes, 2: Evidence from crustal xenoliths at scoria cones and maars. *J Volcanol Geotherm Res.* **223–224,** 47–63 (2012).

59. Sottili, G., Palladino, D.M., Gaeta, M. & Masotta, M. Origins and energetics of maar volcanoes: examples from the ultrapotassic Sabatini Volcanic District (Roman Province, Central Italy). *Bull Volcanol.* **74,** 163–186 (2011).

60. Ross, P.-S., Delpit, S., Haller, M.J., Németh, K. & Corbella, H. Influence of the substrate on maar–diatreme volcanoes — an example of a mixed setting from the Pali Aike volcanic field, Argentina. *J Volcanol Geotherm Res.* **201,** 253–271 (2011).

61. Gençalioğlu-Kuşcu, G., Atilla, C., Cas, R.A.F. & Kuşcu, İ. Base surge deposits, eruption history, and depositional processes of a wet phreatomagmatic volcano in Central Anatolia (Cora Maar). *J Volcanol Geotherm Res.* **159,** 198–209 (2007).

62. Tchamabé, B. *et al.* Eruptive history of the Barombi Mbo Maar, Cameroon Volcanic Line, Central Africa: constraints from volcanic facies analysis. *Open Geosciences.* **5,** 480–496 (2013).

63. Tchamabé, B. *et al*. Temporal evolution and growth of the Barombi Mbo Maar (Cameroon): constraint from juvenile pyroclast distributions. in *5th International Maar Conference, Abstracts Volume: Juriquilla, Qro., México, Universidad Nacional Autónoma de México, Centro de Geociencias* (eds. Carrasco-Núñez, G., Aranda-Gómez, J.J., Ort, M.H. & Silva-Corona, J.J.) 82–83 (2014).

64. Tchamabé, B. *et al.* Towards the reconstruction of the shallow plumbing system of the Barombi Mbo Maar (Cameroon) implications for diatreme growth processes of a polygenetic maar volcano. *J Volcanol Geotherm Res.* **301,** 293–313 (2015).

65. Freundt, A., Hartmann, A., Kutterolf, S. & Strauch, W. Volcaniclastic stratigraphy of the Tiscapa maar crater walls (Managua, Nicaragua): implications for volcanic and seismic hazards and Holocene climate changes. *Int J Earth Sci.* **99,** 1453–1470 (2010).

66. Valentine, G.A. & Groves, K.R. Entrainment of country rock during basaltic eruptions of the Lucero Volcanic Field, New Mexico. *J Geol.* **104,** 71–90 (1996).

67. Womer, M.B., Greely, R. & King, J.S. The geology of split butte — a maar of the south-central snake river plain, Idaho. *Bull Volcanol.* **43,** 453–471 (1980).

68. Amin, J. & Valentine, G.A. Compound maar crater and co-eruptive scoria cone in the Lunar Crater Volcanic Field (Nevada, USA). *J Volcanol Geotherm Res.* **339,** 41–51 (2017).

69. Gevrek, A.İ. & Kazanci, N. A Pleistocene, pyroclastic-poor maar from central Anatolia, Turkey: influence of a local fault on a phreatomagmatic eruption. *J Volcanol Geotherm Res.* **95,** 309–317 (2000).

70. Tchamabé, B., Carrasco-Núñez, G., Miggins, D.P. & Németh, K. Late Pleistocene to Holocene activity of Alchichica maar volcano, eastern Trans-Mexican Volcanic Belt. *J South Am Earth Sci.* **97,** 102404 (2020).

71. Isaia, R. *et al.* Stratigraphy, structure, and volcano-tectonic evolution of Solfatara maar-diatreme (Campi Flegrei, Italy). *Geol Soc Am Bull.* **127,** 1485–1504 (2015).

72. Jordan, S.C., Cas, R.A.F. & Hayman, P.C. The origin of a large (>3km) maar volcano by coalescence of multiple shallow craters: Lake Purrumbete maar, southeastern Australia. *J Volcanol Geotherm Res.* **254,** 5–22 (2013).

73. van den Hove, J.C., Ailleres, L., Betts, P.G. & Cas, R.A.F. Subsurface structure of a large basaltic maar volcano examined using geologically constrained potential field modelling, Lake Purrumbete Maar, Newer Volcanics Province, southeastern Australia. *J Volcanol Geotherm Res.* **304,** 142–159 (2015).

74. Németh, K., Martin, U. & Harangi, S. Miocene phreatomagmatic volcanism at Tihany (Pannonian Basin, Hungary). *J Volcanol Geotherm Res.* **111,** 111–135 (2001).

75. Ngwa, C.N., Suh, C.E. & Devey, C.W. Phreatomagmatic deposits and stratigraphic reconstruction at Debunscha Maar (Mt Cameroon volcano). *J Volcanol Geotherm Res.* **192,** 201–211 (2010).

76. Oms, O. *et al*. Structure of the Pliocene Camp dels Ninots maar-diatreme (Catalan Volcanic Zone, NE Spain). *Bull Volcanol.* **77,** 1–13 (2015).

77. Pittari, A., Briggs, R.M. & Bowyer, D.A. Subsurface geology, ancient hydrothermal systems and crater excavation processes beneath Lake Rotomahana: evidence from lithic clasts of the 1886AD Rotomahana Pyroclastics. *J Volcanol Geotherm Res.* **314,** 110–125 (2016).

78. Sarrionandia, F., Carracedo Sánchez, M., Arostegui, J. & Gil Ibarguchi, J.I. Formation of composite pyroclasts by welding inside a lithic-rich mafic eruption column (Los Marteles Caldera, Canary Islands). *J Volcanol Geotherm Res.* **308,** 99–112 (2015).

79. White, J.D.L. & Schmincke, H.-U. Phreatomagmatic eruptive and depositional processes during the 1949 eruption on La Palma (Canary Islands). *J Volcanol Geotherm Res.* **94,** 283–304 (1999).

80. Mattsson, H.B., Höskuldsson, Á. & Hand, S. Crustal xenoliths in the 6220 BP Sæfell tuff-cone, south Iceland: evidence for a deep, diatreme-forming, Surtseyan eruption. *J Volcanol Geotherm Res.* **145,** 234–248 (2005).

81. Fierstein, J. & Hildreth, W. Eruptive history of the Ubehebe Crater cluster, Death Valley, California. *J Volcanol Geotherm Res.* **335,** 128–146 (2017).

82. Pardo, N. *et al.* The ∼1245 yr BP Asososca maar eruption: the youngest event along the Nejapa–Miraflores volcanic fault, Western Managua, Nicaragua. *J Volcanol Geotherm Res.* **184,** 292–312 (2009).

83. Pardo, N., Avellán, D.R., Macías, J.L., Scolamacchia, T. & Rodríguez, D. The ∼1245 yr BP Asososca maar: new advances on recent volcanic stratigraphy of Managua (Nicaragua) and hazard implications. *J Volcanol Geotherm Res.* **176**, 493–512 (2008).

84. Ort, M.H. & Carrasco-Núñez, G. Lateral vent migration during phreatomagmatic and magmatic eruptions at Tecuitlapa Maar, east-central Mexico. *J Volcanol Geotherm Res.* **181,** 67–77 (2009).

85. Self, S., Kienle, J. & Huot, J.-P. Ukinrek Maars, Alaska, II. Deposits and formation of the 1977 craters. *J Volcanol Geotherm Res.* **7,** 39–65 (1980).

86. Valentine, G.A., Shufelt, N.L. & Hintz, A.R.L. Models of maar volcanoes, Lunar Crater (Nevada, USA). *Bull Volcanol.* **73,** 753–765 (2011).

87. Aranda-Gómez, J. & Luhr, J. Origin of the Joya Honda maar, San Luis Potosí, México. *J Volcanol Geotherm Res.* **74,** 1–18 (1996).

88. Pedrazzi, D., Bolós, X., Barde-Cabusson, S. & Martí, J. Reconstructing the eruptive history of a monogenetic volcano through a combination of fieldwork and geophysical surveys: the example of Puig d’Àdri (Garrotxa Volcanic Field). *J Geol Soc, London.* **173,** 875–888 (2016).

89. Harvey, S. *et al.* Geology and evaluation strategy of the Star and Orion South kimberlites, Fort à la Corne, Canada. *Lithos*. **112,** 47–60 (2009).

90. Zonneveld, J.-P. *et al.* Sedimentologic and stratigraphic constraints on emplacement of the Star Kimberlite, east–central Saskatchewan. *Lithos.* **76,** 115–138 (2004).

91. Leckie, D.A. *et al.* Emplacement and reworking of Cretaceous, diamond-bearing, crater facies kimberlite of central Saskatchewan, Canada. *Geol Soc Am Bull.* **109(8),** 1000–1020 (1997).

92. Brown, R.J. *et al.* Eruption of kimberlite magmas: physical volcanology, geomorphology and age of the youngest kimberlitic volcanoes known on earth (the Upper Pleistocene/Holocene Igwisi Hills volcanoes, Tanzania). *Bull Volcanol.* **74,** 1621–1643 (2012).
